# Genome editing HLA alleles for a pilot immunocompatible hESC line in a Chinese hESC bank for cell therapies

**DOI:** 10.1111/cpr.13471

**Published:** 2023-05-17

**Authors:** Tian‐Tian Ji, Shuai‐Shuai Niu, Ming‐Hui Fang, Ling‐Xue Xu, Xin Wang, Jun Zou, Fei Xu, Meng Zhang, Rui Niu, Jun Wu, Lei Wang, Zhi‐Kun Li, Yong‐Guang Yang, Jie Hao, Wei Li, Zheng Hu, Qi Zhou

**Affiliations:** ^1^ State Key Laboratory of Stem Cell and Reproductive Biology Institute of Zoology, Chinese Academy of Sciences Beijing China; ^2^ University of Chinese Academy of Sciences Beijing China; ^3^ National Stem Cell Resource Center Institute of Zoology, Chinese Academy of Sciences Beijing China; ^4^ Beijing Institute for Stem Cell and Regenerative Medicine Beijing China; ^5^ Institute for Stem Cell and Regenerative Medicine Chinese Academy of Sciences Beijing China; ^6^ Key Laboratory of Organ Regeneration & Transplantation of Ministry of Education The First Hospital of Jilin University Jilin China; ^7^ National‐Local Joint Engineering Laboratory of Animal Models for Human Diseases Jilin China; ^8^ Savaid Medical School University of Chinese Academy of Sciences Beijing China

## Abstract

Robust allogeneic immune reactions after transplantation impede the translational pace of human embryonic stem cells (hESCs)‐based therapies. Selective genetic editing of human leucocyte antigen (HLA) molecules has been proposed to generate hESCs with immunocompatibility, which, however, has not been specifically designed for the Chinese population yet. Herein, we explored the possibility of customizing immunocompatible hESCs based on Chinese HLA typing characteristics. We generated an immunocompatible hESC line by disrupting *HLA‐B*, *HLA‐C*, and *CIITA* genes while retaining HLA‐A*11:01 (HLA‐A*11:01‐retained, HLA‐A11^R^), which covers ~21% of the Chinese population. The immunocompatibility of HLA‐A11^R^ hESCs was verified by in vitro co‐culture and confirmed in humanized mice with established human immunity. Moreover, we precisely knocked an inducible caspase‐9 suicide cassette into HLA‐A11^R^ hESCs (iC9‐HLA‐A11^R^) to promote safety. Compared with wide‐type hESCs, HLA‐A11^R^ hESC‐derived endothelial cells elicited much weaker immune responses to human HLA‐A11^+^ T cells, while maintaining HLA‐I molecule‐mediated inhibitory signals to natural killer (NK) cells. Additionally, iC9‐HLA‐A11^R^ hESCs could be induced to undergo apoptosis efficiently by AP1903. Both cell lines displayed genomic integrity and low risks of off‐target effects. In conclusion, we customized a pilot immunocompatible hESC cell line based on Chinese HLA typing characteristics with safety insurance. This approach provides a basis for establishment of a universal HLA‐A^R^ bank of hESCs covering broad populations worldwide and may speed up the clinical application of hESC‐based therapies.

## Graphical abstract

Here, we first selected an human embryonic stem cell (hESC) line with homozygous human leucocyte antigen (HLA)‐A*11:01 allele, the most popular HLA‐I allele of the Chinese population, then generated a hypoimmunogenic hESC line by disrupting *HLA‐B*, *HLA‐C*, and *CIITA* genes while retaining HLA‐A*11:01 (named as HLA‐A11^R^ hESCs), covering ~21% of the Chinese population. The immunocompatibility of the HLA‐A11^R^ hESCs was verified by in vitro co‐culture assays and confirmed in humanized mice with an established human immune system. Moreover, to address safety issues, we precisely knocked a clinical trial‐grade drug‐induced caspase‐9 suicide cassette into HLA‐A11^R^ hESCs, generating iC9‐HLA‐A11^R^ hESCs, which were efficiently induced to apoptosis by AP1903. In summary, we customized an immunocompatible hESC cell line based on the HLA typing characteristics of the Chinese population with safety insurance, which provided the basis for the establishment of an HLA‐A^R^ bank of hESCs covering broader populations in the world, speeding up the clinical application of a range of hESC‐based therapies.

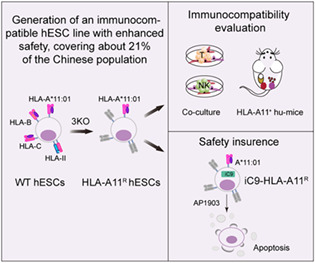

## 1 INTRODUCTION

Human embryonic stem cells (hESCs) are broadly known as a valuable source of regenerative medicine through cell therapy. With the advances in protocols for hESC differentiation, hESC‐derived cells are emerging at a larger scale for the treatment of various diseases in clinical trials,^1^, ^2^ but their broad use is limited by the allogeneic immune rejection. Various solutions have been proposed for the problem of rejection, including human leucocyte antigen (HLA)‐typed hPSC banks,^3^, ^4^ or iPSC banks derived from each patient,^5^ but these approaches require multiple cell lines with high costs and labor. Using immunosuppressants is an alternative to overcoming immune incompatibility but often comes with a high potential for side effects and toxicity.^6^ Therefore, the generation of immunocompatible cells is expected to advance cellular therapies to the clinic. Recent developments of the CRISPR/Cas9 gene editing technology in combination with hESCs differentiation processes have made it accessible.

Allogeneic immune rejection is caused mainly by mismatches of HLAs between the donor and recipient. Recently, several engineering strategies to reduce immune rejection have emerged by modification of HLA molecules.^7^, ^8^, ^9^ Disruption of all HLA‐I (classical HLA‐A, HLA‐B, HLA‐C and non‐classical HLA‐E, HLA‐F, HLA‐G) and HLA‐II (HLA‐DR, HLA‐DQ, HLA‐DP) by knocking out *B2M* and *CIITA* genes^10^, ^11^, ^12^ results in reduced immune surveillance^13^ and NK cell‐mediated killing because of a lack of ligands for the inhibitory receptors of NK cells.^12^, ^14^ To overcome these barriers, overexpression of HLA‐I (e.g., HLA‐E,^15^ HLA‐G,^16^, ^17^ and HLA‐A^18^) or CD47^19^ on the surface of hPSCs lacking HLA‐I and HLA‐II may be a significant attempt. However, whether transgenes can be expressed consistently and stably after hESC differentiation or transplantation into the body is a question worth considering, not to mention that they may only resist partial NK cell killing if they are expressed. Compared with the approaches mentioned above, a strategy based on selective HLA gene editings, such as retention of one classical HLA‐I locus and disruption of the other two while retaining non‐classical HLA‐I (the single retention strategy) may be more desirable from the standpoints of compatibility and surveillance of the immune system. In accordance with the philosophy of this strategy, seven HLA‐C‐retained iPSC lines combined with *CIITA* knockout could cover ~95% of the Japanese population.^20^, ^21^ An HLA‐A*02‐retained hESC line has an average prevalence of 27% in 102 countries,^22^ but the frequency varies widely by geography, relatively high in white populations.^23^ However, the single retention strategy to produce immunocompatible hESCs has not been proposed for the Chinese population. In China, the most highly prevalent allele is HLA‐A*11:01, which covers ~21% of the Chinese population. In addition, more than 95% of the Chinese population could be covered by just 14 highly prevalent HLA‐A‐retained hESC lines.^24^ Therefore, we hypothesized we could customize an immunocompatible hESC line based on the HLA typing characteristics of the Chinese population using an HLA‐A*11:01‐retained strategy, which may provide a theoretical basis for establishment of an HLA‐A^R^ bank of 14 hESC lines covering 95% of the Chinese population and broad populations around the world.

Here, following the selection of homozygous HLA‐A*11:01 hESCs as parental cells, we generated an immunocompatible hESC line based on Chinese HLA typing by deleting *HLA‐B*, *HLA‐C*, and *CIITA* genes while retaining HLA‐A*11:01 and non‐classical HLA‐I molecules. HLA‐A11^R^ hESC‐derived endothelial cells (ECs) escaped HLA‐A11‐matched T‐cell killing but also did not induce activation of NK cells in vitro. HLA‐A11^R^ hESCs and their derivatives could reliably evade human immune responses in humanized mice with an HLA‐A11^+^ functional human immune system. Furthermore, an inducible caspase‐9 system was installed into HLA‐A11^R^ hESCs because of safety concerns, which were induced to commit suicide efficiently by the dimerizer agent rimiducid (AP1903). Moreover, the genomic integrity of engineered hESCs and risks of off‐target were assessed. Taken together, we customized an immunocompatible hESC line with safety insurance based on Chinese HLA typing, which could be the basis for establishment of an HLA‐A^R^ hESC bank covering broad populations around the world, accelerating the progression of translation‐to‐clinic of hESCs for a variety of hESC‐based therapies.

## 2 MATERIALS AND METHODS

### 2.1 Estimation of HLA allele frequency

The Chinese HLA data query website (http://cmms.dnaday.cn/index.jsp?local=zh_CN) was used to estimate the frequency of HLA‐A*11:01 and the frequency order of HLA‐A, ‐B, ‐C, ‐DR, ‐DQ typing in China (Table S7 and S8). Data for HLA‐A*11:01 allele frequency in more populations and regions of the world were extracted from The Allele Frequency Net Database (http://www.allelefrequencies.net; Table S9). The HLA allele sequence was referenced from IPD‐IMGT/HLA database (https://www.ebi.ac.uk/ipd/imgt/hla/alleles/).

### 2.2 Generation of HLA‐A11^R^ hESCs

The wild‐type (WT) hESC line with homozygous HLA‐A*11:01 (coded as C0101078) used in the study was provided by National Stem Cell Resource Center (NSCRC) with strict quality tests. Human ESCs were grown on VTN‐N (Gibco, A14700) pre‐coated plates and cultured in complete Essential 8™ Medium (Gibco, A1517001). For passaging, hESCs were routinely dissociated with 0.5 mM EDTA (Invitrogen, 15575020) and replated in fresh complete medium supplemented with 10 μM Y27632 (MedChemExpress, HY‐10583). The WT hESCs were single‐cell dissociated with TrypLE (Life Technology, 12563029) and electroporated using 4D‐Nucleofector (Lonza, 4D‐Nucleofector™ X Unit) following the manufacturer's protocol. 1 × 10^6^ hESCs were transfected with a mixture of 4 μg GFP‐Cas9 plasmid and 2 μg of gRNAs (Table S1) in 100 μL Primary P3 buffer (Lonza, V4XP‐3032) and the electroporation condition program CA‐137. Two days After electroporation, cells were single‐cell dissociated, filtered through 40 μm‐cell strainers, and GFP‐positive sorted by MoFlo XDP Cell Flow Sorter (Beckman Coulter), then seeded into 6‐well plates at a density of 50 cells/cm^2^ and grow 8–10 days. Single clones were picked using a Pasteur pipette and genotyped by polymerase chain reaction (PCR).

### 2.3 Generation of iC9‐HLA‐A11^R^ hESCs

1 × 10^6^ HLA‐A11^R^ hESCs were electroporated with a mixture of 4 μg hAAVS1‐sited integrated donor plasmid (Homology Arm Left‐CAG‐iCaspase‐9^25^‐T2A‐BSD‐SV40poly(A)‐Homology Arm Right), 2 μg GFP‐Cas9 plasmid and 1 μg gRNA. After electroporation, cells were seeded into 6‐well plates at a density of 10,000 cells/cm^2^. The cells positive for insertion were selected with Blasticidin S HCl selection (Gibco, A1113903), and colonies were then picked and genotyped by PCR. All primers used for PCR are listed in Table S2.

### 2.4 Pluripotency analysis by immunofluorescence staining

Various hESC lines were dissociated with 0.5 mM EDTA for 5 min and passaged onto VTN‐coated 24‐well plates in E8 complete Medium containing Y27632. Wells of the 24‐well plate were pre‐plated 14 mm round glass slides before VTN‐N coating. On Day 2 after plating, aspirate the culture medium from the vessel containing hESCs, and rinse the vessel twice with Dulbecco's phosphate‐buffered saline (DPBS) without Calcium and Magnesium. Cells were fixed with 4% PFA for 30 min, permeabilized, and blocked with 0.3% Triton X‐100 and 2% BSA in DPBS for 1 h at room temperature. Cells were then incubated in 2% BSA containing Rabbit anti‐Nanog antibody (Abcam, ab109250, 1:100), rabbit anti‐Sox2 antibody (CST, no. 23064, 1:300), Mouse anti‐Oct4 antibody (CiteAb, sc‐5279, 1:250) and mouse anti‐SSEA4 antibody (CiteAb, MAB4303, 1:250) at 4°C overnight. Primary antibody staining was followed by labelling with Alexa Fluor 488‐conjugated donkey anti‐rabbit (A21206, 1:400), Alexa Fluor 488–conjugated donkey anti‐mouse (A21202, 1:400) secondary antibodies, and Hoechst 33342 (H3570, 1:1000) from Invitrogen. Fluorescence images were taken with the Leica TCS Sp8 confocal microscopes.

### 2.5 Detection of the expression of HLA molecules by flow cytometry

The expression levels of HLA‐I (Anti‐human HLA‐ABC, BD bioscience, 555552), HLA‐A11 (Antibodies, ABIN2282775), and HLA‐BC (eBioscience, 17‐5935‐42) were detected on hESCs after 48 h of interferon (IFN)‐γ stimulation through flow cytometry (FCM). The expression of HLA‐DR (Biolegend, 327022) was detected on hESC‐derived ECs after 48 h of IFN‐γ (Peprotech, AF‐300‐02) stimulation through FCM. Cells were incubated with recommended antibodies on ice for 20 min in the dark.

### 2.6 T‐cell proliferation assay

As target cells, hESC‐derived ECs were plated into gelatin‐coated 48‐well plates at a concentration of 1 × 10^5^/well, and their HLA‐I/II expression would be elevated after 48 h of IFN‐γ stimulation. The isolated CD3^+^ T cells were labelled by CFSE according to the manufacturer's instructions. After washing the IFN‐γ‐stimulated hESC‐ECs twice with DPBS, they were co‐incubated with CFSE‐labelled CD3^+^ T cells at a 1:1 ratio in 48‐well plates for 5 days in full 1640 supplemented with 20 U/mL IL‐2. The negative control was set as T cells cultured for 5 days without target cells, and the positive control was set as T cells treated with the human CD3^+^/CD28^+^ T‐cell activator (Stemcell, 10981) for 5 days.

### 2.7 T‐cell activation assay

As target cells, hESC‐derived ECs were plated into gelatin (Sigma, V90086)‐coated 48‐well plates at a concentration of 1 × 10^5^/well, and their HLA‐I/II expression would be elevated after 48 h of IFN‐γ stimulation. After washing the IFN‐γ‐stimulated hESC‐derived ECs twice with DPBS, they were co‐incubated with isolated CD3^+^ T cells at a 1:1 ratio in 48‐well plates. The expression of CD69 (Biolegend, 310914) and CD154 (Biolegend, 310824), activation markers of T cells, were detected on the third and sixth day of co‐incubation, respectively. The negative control was set as T cells cultured without target cells, and the positive control was set as T cells treated with human CD3^+^/CD28^+^ T‐cell activator.

### 2.8 T‐cell killing assay

1 × 10^5^ hESC‐derived ECs (target cells) which were pre‐treated with IFN‐γ for 48 h, and isolated T cells (effector cells) at the 1:1 effector/target ratios were co‐incubated in full 1640 supplemented with 20 U/mL IL‐2 medium for 5 days. The supernatant was collected and analysed by CytoTox 96 Non‐Radioactive Cytotoxicity Assay kit (Promega, G1780) following the manufacturer's instructions. Full 1640 supplemented with 20 U/mL IL‐2 medium was used as background control. T cells or hESC‐derived ECs cultured alone were used as spontaneous lactate dehydrogenase (LDH) release. The maximum LDH release was detected by lysed hESC‐derived ECs at the endpoint.

### 2.9 NK cell degranulation assay

5 × 10^4^ hESC‐derived ECs (target cells) and isolated NK cells (effector cells) at the 1:1 effector/target ratios were co‐incubated in 200 μL full 1640 medium with anti‐hu CD107a (LAMP‐1), FITC (Invitrogen, 11107942) in 96‐well U‐bottom plate. Centrifugation at 300*g* for 5 min to bring effector cells into full contact with target cells before incubation. After 20 h of co‐incubation, the expression of CD107a on NK cells was detected by FCM. NK cells cultured alone were used as the negative control, while ones treated with 10 ng/mL phorbol 12‐myristate 13‐acetate (PMA) (Sigma, P1585) and 1 μg/mL ionomycin (Sigma, I3909) were used as the positive control.

### 2.10 NK cell killing assay

5 × 10^4^ hESC‐derived ECs (target cells) and isolated NK cells (effector cells) at the 3:1, 1:1, 1:3 effector/target ratios were co‐incubated in 200 μL full 1640 medium in 96‐well U‐bottom plate. Centrifugation at 300*g* for 5 min to bring effector cells into full contact with target cells before incubation. After 20 h of co‐incubation, the apoptosis of target cells was quantified using FCM by detecting the positive of 7‐AAD in target cells (Biolegend, 420404). CFSE‐labelled K562 tumour cell line was used as the positive control.

### 2.11 Mouse model

NSG immunodeficient mice: Non obese diabetes (NOD)‐Prkdc^em26Cd52^IL2rg^em26Cd22^/Nju (NOD/SCID IL2rg^−/−^ mice; abbreviated as NSG; also named as NCG) mice were purchased from GemPharmatech Co., Ltd. We maintained them under specific pathogen‐free animal facilities. The female mice were used in experiments at 6–8 weeks of age. Humanized mouse model: HLA‐A11^+^ human foetal thymus and liver tissues of gestational age of 17–20 weeks were obtained from the First Hospital of Jilin University. Humanized mice were constructed by transplantation with human foetal thymic tissue and CD34^+^ haematopoietic stem/progenitor cells as previously described.^26^ Briefly, the 6–8 weeks old female NSG mice were sub‐lethally (1.75 Gy‐irradiated), followed by co‐transplanting of cryopreserved human foetal thymic tissues under the kidney capsule and hCD34^+^ foetal liver cells (1.5–2.0 × 10^5^/each) injection by tail vein. HLA haplotype information of humanized mice was listed in Table S5.

### 2.12 Teratoma assays

4 × 10^6^ WT hESC or HLA‐A11^R^ hESC were mixed with matrigel at a 1:1 ratio in a 100 μL system and separately injected into the subcutaneous right hindlimb of the NSG and humanized mice. Mice were sacrificed 8 weeks later and analysed by the haematoxylin–eosin (H&E) and immunohistochemistry (IHC) staining of parafcfin‐embedded sections.

### 2.13 Transplantation of ECs from WT or HLA‐A11^R^ hESC

Two million WT ECs or HLA‐A11^R^ ECs in a pro‐survival scaffold (100 μL), consisting of 50% (vol/vol) Matrigel (Corning), Recombinant Human vascular endothelial growth factor (VEGF) (75 ng/mL), Recombinant Human basic fibroblast growth factor (bFGF) (300 ng/mL) were injected into subcutaneous bilateral hindlimbs of humanized mice. Mice were sacrificed 3 weeks later and analysed by the H&E and IHC staining of paraffin‐embedded sections.

### 2.14 FCM analysis about animals

Peripheral blood mononuclear cells (PBMCs) and spleen were harvested from humanized mice by density gradient centrifugation by Histopaque‐1077 (Sigma‐Aldrich). Fluorochrome conjugated monoclonal antibodies for human antigens included CD45 (BioLegend, APC‐Cy7, clone HI30), CD45 (BD, BV480, clone HI30), CD3 (BD, PE‐CY7, clone SK7), CD4 (BD, BV750, clone SK3), CD8 (BioLegend, PerCP/Cyanine5.5, clone SK1), CD19 (BD, BB515, clone HIB19), CD33 (BioLegend, BV421, clone P67.6), CD56 (BD, BV650, clone NCAM16.2), CD45RA (BioLegend, BV510, clone HI100), CD45RO (Biolegend, APC‐Cy7, clone UCHL1), anti‐mouse CD45 (BioLegend, Pacific Blue™, clone 30‐F11), and mouse Ter119 (BioLegend, PE‐Dazzle™ 594, clone TER119). All antibodies were obtained from Biolegend (California, USA) and BD (USA), unless otherwise specified. Data were acquired with fluorescence‐activated cell sorting (FACS) Diva on an Aurora flow cytometer (Cytek, Aurora, USA), and analysed with FlowJo software.

### 2.15 Histological analysis

Tissues were harvested and fixed in 10% buffered formalin and embedded in paraffin for H&E staining. Serial sections (2.5 μm) were prepared for IHC examination of human CD4 (ABclonal Technology, clone 1H1R9, 1:500), CD8 (DAKO, clone C8/144B, 1:100), and CD31 (Abcam, ab28364, polyclonal). Immunoreactivity was detected with UltraSensitive TM Streptavidin‐Peroxidase Kit (Mai Xin, KIT‐9710) according to the manufacturer's protocol. The numbers of positively stained cells were assessed using the IHC profiler of Image J (NIH) and all the slides were observed and photographed with laser scanning confocal microscopy (LSM 880) using a 10× objective.

### 2.16 Induction and analysis of apoptosis

Edited hESCs were cultured in E8 medium for 2–4 days in a 24‐well plate when cells cover approximately 80% of the surface area of the culture vessel and then exposed to 10 nM AP1903 (MCE, HY‐16046) for the desired amount of time. The edited hESCs treated with AP1903 were harvested by TrypLE digestion followed by staining with Trypan blue (Gibco, 15250061) and annexin V (AnV; Abcam, ab14147) according to the manufacturer's instructions. Live and dead cells stained with Trypan blue were counted by Countess II (Thermo Fisher Scientific). Cells stained with AnV were quantified and analysed by FCM (BD LSRFortessa) and FlowJo 10.5.3. Cell morphology and number change were also assessed by microscopy at representative time points.

### 2.17 Statistical analyses

Statistical analysis was performed with the GraphPad Prism 8 software, using the two‐tailed unpaired or one‐tailed paired Student's *t*‐test or Mann–Whitney test analysis. The data were presented as mean values ± SEM. A *p*‐value was considered statistically significant in all types of analyses as follows: *p < 0.05, **p < 0.01, ***p < 0.001, ****p < 0.0001, ns: not significant.

## 3 RESULTS

### 3.1 Generation of customized HLA‐A11^R^ hESCs based on the characteristic of Chinese HLA typing

**FIGURE 1:**
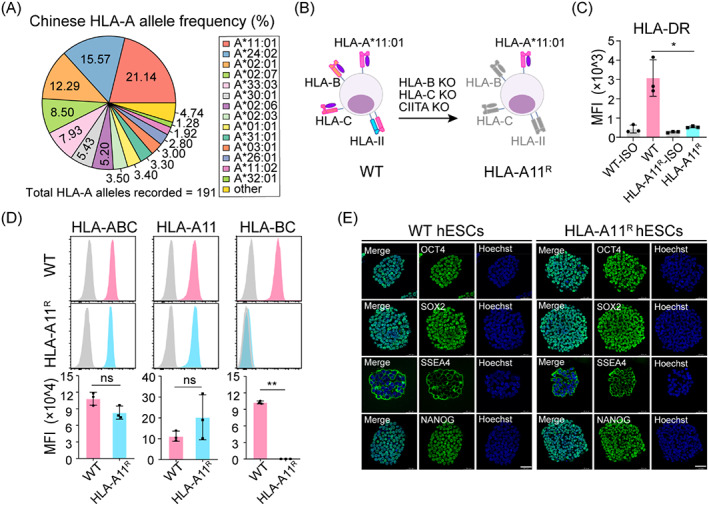
Generation of customized HLA‐A11^R^ human embryonic stem cells (hESCs) based on Chinese human leucocyte antigen (HLA) typing. (A) The 191 known HLA‐A allele frequencies among 169,995 donors in the CMDP registry in China (accessed 2016). (B) Schematic diagram of knockout of *HLA‐B*, *HLA‐C*, and *CIITA* genes to generate an immunocompatible cell line: HLA‐A11^R^ hESCs. (C) HLA‐DR expression in endothelial cells differentiated from WT or HLA‐A11^R^ hESCs upon interferon (IFN‐γ) stimulation. (D) Flow cytometry analysis of HLA‐ABC, HLA‐A11, and HLA‐BC expression in wild‐type (WT) or HLA‐A11^R^ hESCs. Both hESC lines were treated with IFN‐γ for 48 h before staining with the indicated antibodies. (E) Immunofluorescence staining of WT hESCs and HLA‐A^R^ hESCs for the pluripotency markers, OCT4, SOX2, SSEA4, and NANOG. DNA was stained with Hoechst. Scale bar, 48.3 μm.

We analysed the data of HLA‐A, HLA‐B, and HLA—C allele frequencies among the 169,995 donors in the CMDP registry in China (updated to 2016; Figures 1A, S1A, and Table S7). We found that HLA‐A*11:01 was the most prevalent allele (accounting for ~21% of the Chinese population), and the most popular allele across all regions of China (Figure S1B and Table S8), in addition to being found in 1% to 22% of different populations worldwide (Figure S1C and Table S9). These data suggested the great potential of hESCs, which require only the HLA‐A*11:01 match, as valuable resources for stem cell therapy. To generate immunocompatible cells based on Chinese HLA typing, we used CRISPR/Cas9 technology to knockout *HLA‐B*, *HLA‐C*, and *CIITA* genes following the screening of homozygous HLA‐A*11:01 hESCs as the parental cells from NSCRC, obtaining HLA‐A*11:01‐retained (HLA‐A11^R^) hESCs (Figure 1B). Due to the high homology of HLA genes, we undertook large‐fragment knockouts of *HLA‐B* and *HLA‐C* genes by designing specific gRNAs at each end of the genes with a cleavage efficiency >40% (Table S1). After two rounds of co‐electroporating gRNA/Cas9‐expressing plasmids into WT hESCs, we identified positive PCR‐genotyping products (HLA‐A11^R^ hESCs) in 4.5% of derived clones (Figure S2A and Table S3). Analyses of gel electrophoresis and Sanger sequencing revealed the specific deletion of *HLA‐B*, *HLA‐C*, and *CIITA* genes on HLA‐A11^R^ hESCs (Figure S2B,C). After interferon (IFN)‐γ stimulation for 48 h, the ablation of the proteins of HLA‐B and HLA‐C in HLA‐A11^R^ hESCs was confirmed by FCM (Figure 1D). To demonstrate the destruction of HLA‐II molecules, we further differentiated both WT and HLA‐A11^R^ hESCs into ECs^27^ (WT ECs and HLA‐A11^R^ ECs), which expressed both HLA‐I and HLA‐II following IFN‐γ stimulation in cells without gene editing. Both types of hESC‐derived ECs expressed an equal level of EC markers, CD31 and CD144 (VE‐Cadherin), which suggested that genome editing did not affect the differentiation efficiency of HLA‐A11^R^ hESCs (Figure S3A–D). Deletion of the HLA‐II protein was confirmed by staining of HLA‐DR antibodies using FCM (Figure 1C). To confirm that HLA‐A11^R^ hESCs retained pluripotency, expression of OCT4, SOX2, SSEA4, and NANOG was assessed by immunofluorescence staining in HLA‐A11^R^ hESCs, and was found to be equivalent to that of WT hESCs (Figure 1E). In addition, the pluripotency of HLA‐A11^R^ hESCs was characterized by quantitative real‐time PCR (qRT‐PCR), FCM, and analysis of teratoma tissue (Figure S4A,B,D). In summary, we generated a customized immunocompatible hESC (HLA‐A11^R^) line with normal pluripotency by deleting *HLA‐B*, *HLA‐C*, and *CIITA* genes and retaining homozygous HLA‐A*11:01, covering ~21% of the Chinese population.

### 3.2 HLA‐A11^R^ hESC‐derived ECs inhibited HLA‐A11‐matched T‐cell responses in vitro

**FIGURE 2:**
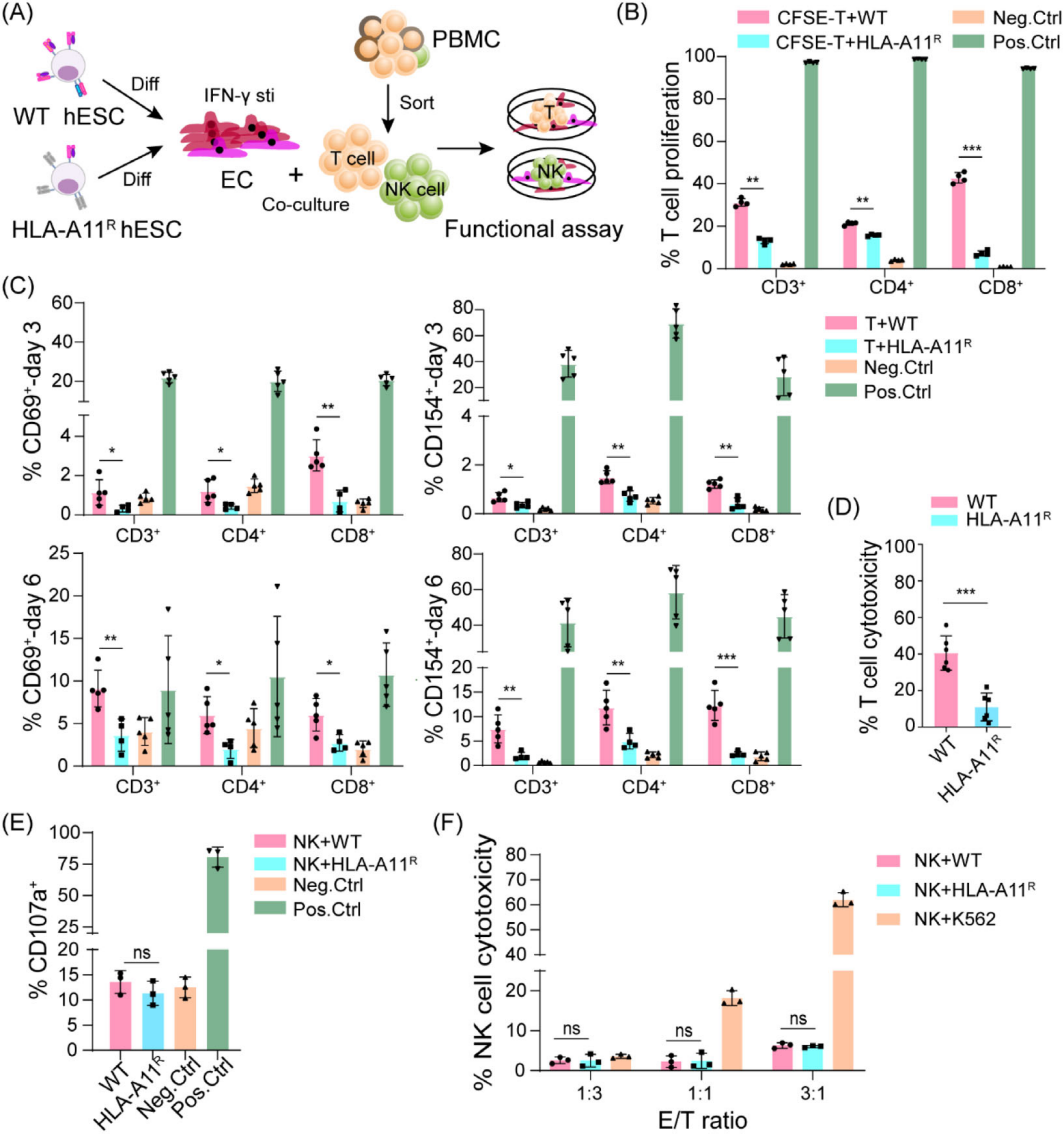
Reduced T cell and NK cell responses against allogeneic human leucocyte antigen (HLA)‐A11^R^ hESC‐derived endothelial cells (ECs) in vitro. (A) The schematic diagram for immunogenicity assay of hESC‐derived ECs. Wild‐type (WT) or HLA‐A11^R^ hESCs were differentiated into ECs, stimulated with interferon (IFN)‐γ, and co‐cultured with T cells or NK cells isolated from PBMCs. Assays for T‐cell activation, T‐cell proliferation, T‐cell cytotoxicity, NK cell degranulation, and NK cytotoxicity were conducted to assess the immune compatibility of HLA‐A11^R^ ECs. (B) Assay to measure T‐cell proliferation with flow cytometry (FCM): the percentage of CFSE^−/low^ cells in CD3^+^, CD4^+^, and CD8^+^ T‐cell populations after co‐culturing with WT or HLA‐A11^R^ ECs. T cells cultured alone were used as the negative control (Neg. Ctrl); T cells activated by CD3 and CD28 antibodies were used as the positive control (Pos. Ctrl). (C) Assay to measure T‐cell activation with FCM: the percentage of CD69^+^ and CD154^+^ cells in CD3^+^, CD4^+^, and CD8^+^ T‐cell populations after co‐culture with WT or HLA‐A11^R^ ECs. T cells cultured alone were used as Neg. Ctrl; T cells activated by CD3 and CD28 antibodies were set as Pos. Ctrl. (D) Scatterplot displaying the percentage of T‐cell cytotoxicity against WT or HLA‐A11^R^ ECs. (E) Scatterplot of the percentage of CD3^−^CD56^+^CD107a^+^ cells as a readout for NK cell degranulation against WT or HLA‐A11^R^ ECs. NK cells cultured alone were used as Neg. Ctrl; NK cells treated with PMA/ionomycin were set as Pos. Ctrl. (F) Assay to measure the cytotoxicity of NK cells with FCM: the percentage of 7‐AAD^+^ in WT or HLA‐A11^R^ ECs co‐incubated with NK cells isolated from a single donor. CFSE‐labelled K562 tumour cell line was set as Pos. Ctrl.

To investigate whether HLA‐A11^R^ hESCs enabled to a reduction of immune responses in vitro, we co‐cultured WT or HLA‐A11^R^ ECs with allogeneic T cells from PBMCs of healthy donors, who had matched HLA‐A*11:01 but other HLA alleles that were mismatched (Figure 2A). To assess the T‐cell proliferation, CFSE‐labelled allogeneic CD3^+^ T cells were co‐cultured with IFN‐γ‐pre‐treated WT or HLA‐A11^R^ ECs for 5 days. The percentage of proliferative CD3^+^ T cells was lower when incubated with HLA‐A11^R^ ECs compared with WT ECs, consistent with proliferative CD4^+^ and CD8^+^ T‐cell subpopulations (Figures 2B and S5A). However, CD8^+^ cytotoxic T cells exhibited a more pronounced reduction in proliferation, suggesting that HLA‐A11^R^ ECs were more able to suppress CD8^+^ T‐cell proliferation than other T subpopulations. In summary, HLA‐A11^R^ ECs could hinder the proliferation of HLA‐A11‐matched CD3^+^ T cells significantly, especially the CD3^+^CD8^+^ T‐cell population.

To confirm expression of the markers of T‐cell activation after co‐culture with different hESC‐derived ECs, WT and HLA‐A11^R^ ECs were pre‐treated with IFN‐γ and co‐cultured subsequently with CD3^+^ T cells for 3 or 6 days. We noticed a lower percentage of CD69^+^ and CD154^+^ T cells in co‐culture with HLA‐A11^R^ ECs compared with WT ECs, the same as CD4^+^ and the CD8^+^ T‐cell subpopulations (Figures 2C and S5B,C). To study T‐cell cytotoxicity upon co‐culture with different types of ECs, LDH release was measured after co‐culture with WT or HLA‐A11^R^ ECs for 5 days. T‐cell cytotoxicity against HLA‐A11^R^ ECs was ~10.31%, which was significantly lower than that of the WT ECs group (40.65%; Figure 2D), thereby suggesting that T‐cell cytotoxicity was suppressed by HLA‐A11^R^ ECs, and consistent with the results of the assay for CD8^+^ T‐cell proliferation. Altogether, these data suggested that HLA‐A11^R^ ECs suppressed the response of allogeneic HLA‐A11‐matched T cells in terms of proliferation, activation, and cytotoxicity.

### 3.3 HLA‐A11^R^ hESC‐derived ECs evaded NK cell responses in vitro

Failure of inhibitory receptors to bind to allogeneic HLA molecules may contribute to NK cell‐mediated destruction of allogeneic donor cells. We wished to assess if the strategy of retaining HLA‐A11 expression in combination with non‐classical HLA‐I was sufficient to resist NK cell‐mediated activity. Hence, we compared the degranulation ability and cytotoxicity of NK cells against WT or HLA‐A11^R^ ECs using a co‐culture system in vitro (Figure 2A). First, we conducted degranulation assays by quantifying cell‐surface expression of CD107a, a protein transferred from the intracell to the cell surface when NK cells are activated to release cytotoxic granules. No differential surface expression of CD107a on NK cells was detected when NK cells were exposed to WT or HLA‐A11^R^ ECs (Figures 2E and S5D), which suggested that retention of HLA‐A11 expression in combination with non‐classical HLA‐I could aid inhibition of NK cell activation. Then, we quantified NK cell cytotoxicity against WT or HLA‐A11^R^ ECs by detecting apoptotic target cells. NK cell cytotoxicity against HLA‐A11^R^ ECs was not significantly different from WT ECs in terms of the E/T ratio (Figure 2F), in accordance with NK cell degranulation. In conclusion, HLA‐A11^R^ hESC‐derived ECs could evade the activation and cytotoxicity of NK cells in vitro.

### 3.4 HLA‐A11^R^ hESCs were compatible with HLA‐A11 ^+^ human immunity in vivo

**FIGURE 3:**
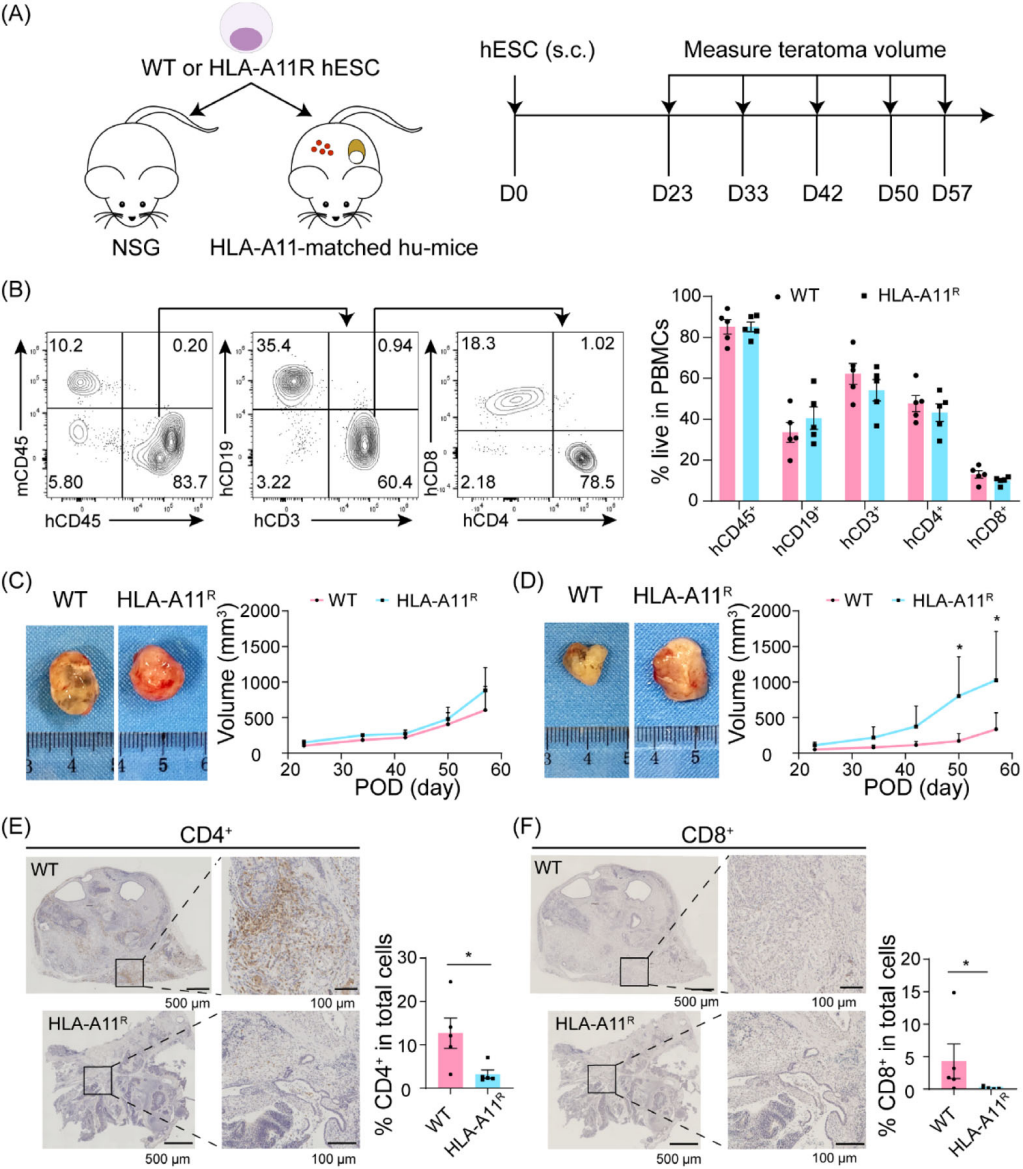
Evasion of human immune surveillance by human leucocyte antigen (HLA)‐A11^R^ hESC teratomas in humanized mice with established HLA‐A11^+^ human immune system. (A) Schema profile of the study. (B) Humanized mice (*n* = 10) were randomly selected for subcutaneous inoculation with 4 × 10^6^ wild‐type (WT; *n* = 5) or HLA‐A11^R^ human embryonic stem cells (hESCs; *n* = 5) at Week 12 after humanization. Non‐humanized NSG mice were set as controls. Representative flow cytometry (FCM) profiles (left) and summarized data (right) of the ratios of distinct subsets of human immune cells in PBMCs at Week 12. (C) Representative macroscopic appearance (left, collected at Day 57) and growth curve (right) of WT (*n* = 4) and HLA‐A11^R^ (*n* = 4) teratomas in NSG mice. (D) Representative macroscopic appearance (left, collected at Day 57) and growth curve (right) of WT (*n* = 5) and HLA‐A11^R^ (*n* = 5) teratomas in humanized mice. (E) Immunohistochemical photographs (left) and summarized data (right) of human CD4^+^ T‐cell infiltration in WT (up) and HLA‐A11^R^ (down) teratomas formed in humanized mice. (F) Immunohistochemical photographs (left) and summarized data (right) of human CD8^+^ T‐cell infiltration in WT (up) and HLA‐A11^R^ (down) teratomas formed in humanized mice.

To simulate HLA‐matched clinical experimental therapy using hESCs and identify the immunogenicity of HLA‐A11^R^ hESCs in mice, we constructed HLA‐A11‐matched humanized mice by transplanting human foetal thymic tissue and CD34^+^ haematopoietic stem/progenitor cells.^26^, ^28^, ^29^ High levels of human immune cells composed of human T cells (including CD4^+^ T cells and CD8^+^ T cells) and B cells were detected 12 weeks post‐transplantation, and subsequent experiments were conducted in random groups (Figure 3A,B). WT and HLA‐A11^R^ hESCs were implanted subcutaneously in NSG mice and humanized mice to assess immunogenicity, and teratoma size was measured periodically. There was no difference in teratoma size or growth curves in NSG mice between WT hESCs and HLA‐A11^R^ hESCs (Figure 3C). The teratomas formed by WT and HLA‐A11^R^ hESCs in humanized mice both tended to grow. However, the growth rate of teratomas derived from HLA‐A11^R^ hESCs was faster than those derived from WT hESCs, and macroscopically, teratomas had a big difference in size may be due to the fact that HLA‐A11^R^ hESCs in humanized mice are less attacked by the immune system (Figure 3D). Humanized mice were euthanized 8 weeks after transplantation of WT or HLA‐A11^R^ hESCs. Teratoma transplantation did not affect the composition of subsets of human immune cells significantly (Figure S6A) or the number of CD45RO^+^ memory cells within human T (CD4^+^ T or CD8^+^ T) cells (Figure S6B) in PBMCs or spleens in these mice. Analysis of serial sections of the teratomas formed by WT or HLA‐A11^R^ hESCs in humanized mice indicated that WT‐derived teratomas were infiltrated by massive number of human CD4^+^ (~12%) and CD8^+^ (~4%) T cells. In contrast, much fewer human CD4^+^ (~3%) and CD8^+^ (~1%) T cells infiltrated in HLA‐A11^R^‐derived teratomas in humanized mice (Figure 3E,F). These data demonstrated that HLA‐A11^R^ hESCs were compatible with an HLA‐A11^+^ human immune system.

### 3.5 Hypoimmunogencity of HLA‐A11^R^ hESC‐derived ECs under HLA‐A11 ^+^ human immune surveillance in vivo

**FIGURE 4:**
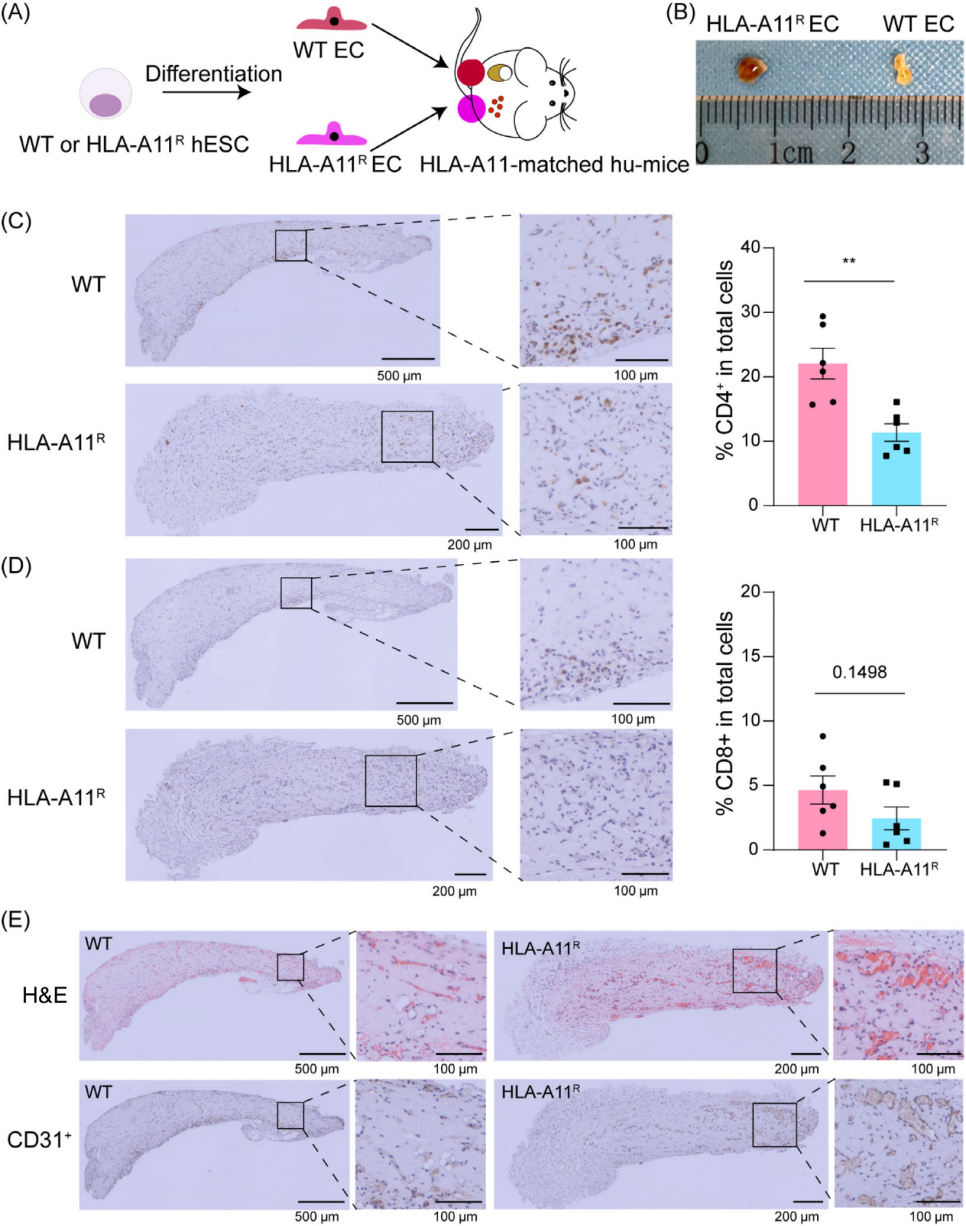
Hypoimmunogenicity of human leucocyte antigen (HLA)‐A11^R^ human embryonic stem cell (hESC)‐derived endothelial cells (ECs) in humanized mice with established HLA‐A11^+^ human immune system. (A) The schema profile of the study was shown. (B) Representative macroscopic appearance (collected at Week 3) of wild‐type (WT) and HLA‐A11^R^ EC‐formed lumps in humanized mice. (C) Immunohistochemical photographs (left) and summarized data (right) of human CD4^+^ T‐cell infiltration in WT (up) and HLA‐A11^R^ (down) ECs in humanized mice (*n* = 6). (D) Immunohistochemical photographs (left) and summarized data (right) of human CD8^+^ T‐cell infiltration in WT (up) and HLA‐A11^R^ (down) ECs in humanized mice (*n* = 6). (E) The panorama of Matrigel plugs from WT ECs and HLA‐A11^R^ ECs stained by haematoxylin–eosin (H&E) and anti‐CD31 antibody.

To further evaluate the immunogenicity of WT and HLA‐A11^R^ hESC‐derived cells, we differentiated hESCs into ECs because of their potential application in the treatment of cardiovascular diseases and ischemic diseases. First, the reconstruction level of the human immune system in HLA‐A11^+^ humanized mice was evaluated by detecting human immune cells 14 weeks post‐transplantation (Figure S6C). Then, WT and HLA‐A11^R^ ECs were separately transplanted into subcutaneous bilateral hindlimbs, and Matrigel plugs were observed 3 weeks post‐transplantation (Figure 4A). Markedly, the macroscopic image of the HLA‐A11^R^ EC plug showed a better blood supply than the WT EC plug (Figure 4B). And, there is no obvious difference in the size of teratomas (Figure S6D). Analysis of serial sections of the Matrigel plugs formed by WT or HLA‐A11^R^ ECs in humanized mice indicated that human CD4^+^ (~22%) and CD8^+^ (~5%) T cells infiltrated with WT ECs. However, the immunogenicity of HLA‐A11^R^ ECs, which infiltrated with human CD4^+^ (~10%) and CD8^+^ (~2%) T cells, appeared to be much weaker than that of WT ECs (Figure 4C,D). Results showing infiltration of CD8^+^ T cells to be less than that of CD4^+^ T cells indicated that matched HLA‐A might attenuate HLA‐I‐mediated CD8^+^ T‐cell responses. Furthermore, HLA‐A11^R^ ECs organized into more structures resembling primitive vascular structures, which occasionally contained erythrocytes than WT ECs according to staining of HE and CD31 (Figure 4E), which suggested the potential of hESC‐derived ECs as therapeutic cells to promote vascular regeneration. Collectively, HLA‐A11^R^ ECs had lower immunogenicity than WT ECs in humanized mice with an established HLA‐A11^+^ human immune system.

### 3.6 Integration of the iC9 suicide cassette into HLA‐A11^R^ hESCs for safety insurance

**FIGURE 5:**
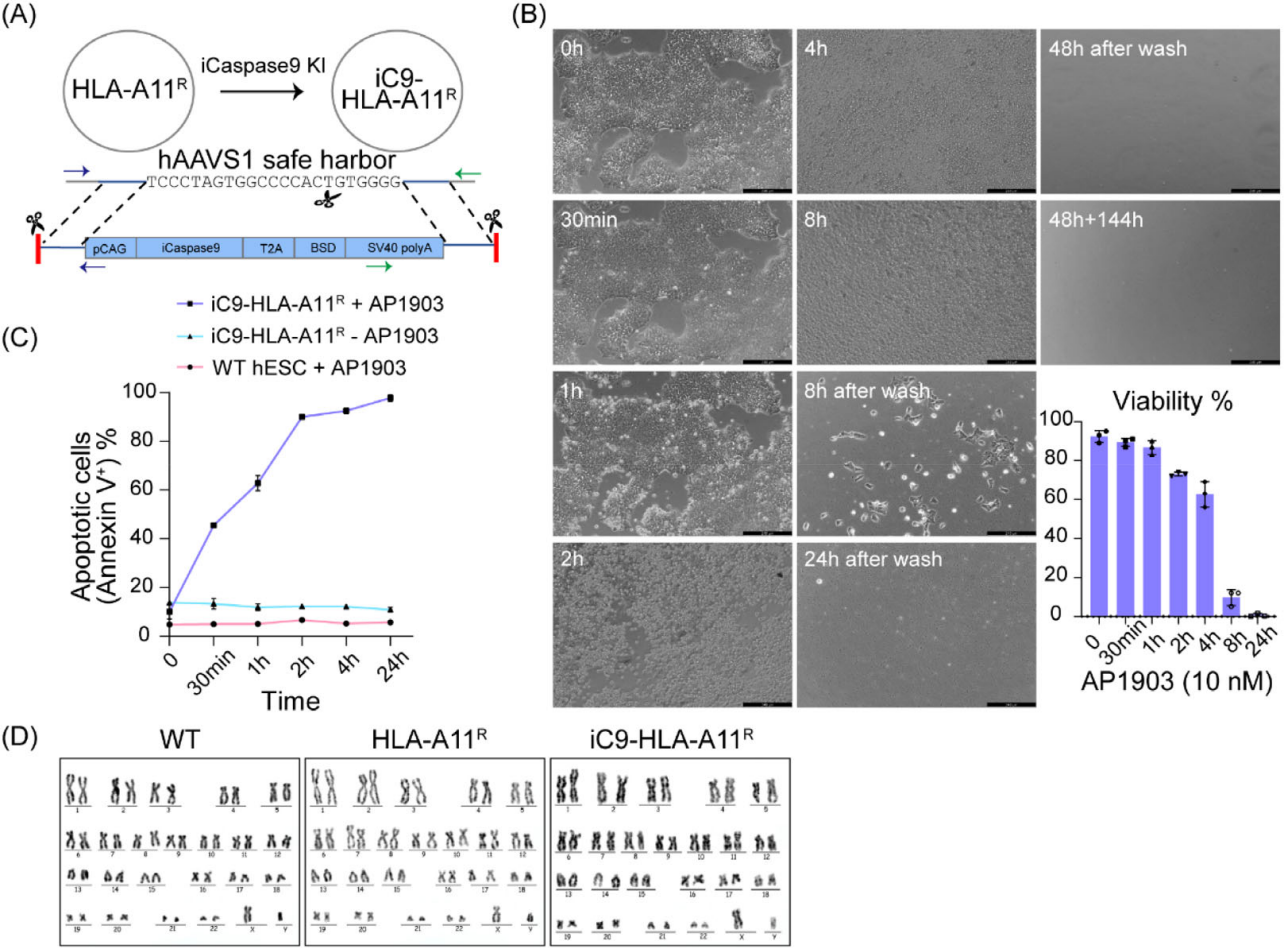
Integration of the iC9 suicide cassette into human leucocyte antigen (HLA)‐A11^R^ human embryonic stem cells (hESCs) for safety insurance. (A) Schematic illustration of precise installation of the inducible caspase‐9 cassette into the AAVS1 safe harbour locus of HLA‐A11^R^ hESCs. Blue and green arrows represent primers of confirmation for iC9‐KI‐left and iC9‐KI‐right, respectively. (B) Representative time point morphologies show AP1903 (10 nM) completely killed iC9‐HLA‐A11^R^ hESCs within 24 h with no cell regrowth until Day 8. Scale bar, 249 μm. Quantifications of killing efficiency at different representative time points by counting the cell viability with trypan blue. (C) FACS analyses of the apoptosis of iC9‐HLA‐A11^R^ and wild‐type (WT) hESC (annexin V) with or without AP1903 (10 nM) treatment at different time points. (D) Karyotype analysis of WT, HLA‐A11^R^, and iC9‐HLA‐A11^R^ hESCs.

To improve the safety of HLA‐A11^R^ hESCs in stem cell‐based therapy, we precisely installed the inducible caspase‐9 suicide system into the AAVS1 safe harbour to generate iC9‐HLA‐A11^R^ hESCs (Figure 5A). After co‐electroporating gRNA/Cas9‐expressing and iC9‐cassette plasmids into HLA‐A11^R^ hESCs, we identified positive PCR‐genotyping products (iC9‐HLA‐A11^R^ hESCs) in 53.3% of genotyped clones (Figure S7A,B and Table S3). First, iC9‐HLA‐A11^R^ hESCs were treated in the presence of AP1903 at different concentrations and different times to explore the optimal concentration of AP1903. Treatment with AP1903 at 10 nM for 8 h caused almost all cells to die, but no difference in cell death (all cells died) was observed if AP1903 at 0.001 to 1000 nM was used for 24 h (Figure S7C). Hence, 10 nM concentration of AP1903 was selected for subsequent apoptosis‐induction experiments. Treatment with AP1903 (10 nM) induced obvious apoptosis of iC9‐HLA‐A11^R^ hESCs within 2 h; Cells became detached from the culture dish, shrank, and degranulated after 24 h (Figure 5B). The killing efficiency at different representative time points was measured by counting the cell viability with trypan blue (Figure 5B): almost no cells were alive after 24 h. In addition, >99% of iC9‐HLA‐A11^R^ hESCs were stained positive with annexin V (apoptosis marker) after treatment with AP1903 for 24 h (Figures 5C and S7F), but this effect was not shown in WT hESCs upon AP1903 treatment, or in iC9‐HLA‐A11^R^ hESCs not treated with AP1903. This distinguishment was not caused by rates of cell proliferation (Figure S7D), but possibly by the relatively high expression of iC9 detected in iC9‐HLA‐A11^R^ hESCs compared with that in WT and HLA‐A11^R^ hESCs (Figure S7E). In summary, precise installation of the iC9 suicide cassette on HLA‐A11^R^ hESCs could eliminate these cells efficiently upon AP1903 treatment.

We also demonstrated that the pluripotency of iC9‐HLA‐A11^R^ hESCs was normal (Figure S4A–C). Additionally, both engineered hESC lines displayed a normal karyotype, consistent with that of WT hESCs (Figure 5D and Table S4). To uncover the mutations that occurred during genome editing and single‐clone cell culture, we conducted whole genome sequencing (WGS)‐based off‐target analysis (Figure S8A). First, the potential off‐target sites of each gRNA were identified by Cas‐OFFinder in the hg38 human genome allowing for up to 5 bp of mismatches (Figure S8B). Except for the widely known NGG, the protospacer adjacent motif (PAM) sequence also included NAG and NGA. The aligned sequences obtained from engineered hESC lines were compared with their parental WT hESCs. Potential off‐target sites based on gRNAs were *SPATA2P1‐RN7SKP6* and *Tango6* genes found in HLA‐A11^R^ hESCs, and the *SP100* gene in iC9‐HLA‐A11^R^ hESCs (Figure S8C). Potential off‐target sites that are not gRNA‐based but related to cancer genes were *Metazoa‐SRP*, *RARA*, and *ACSL6* found in HLA‐A11^R^ hESCs, and *PLCG1* found in iC9‐HLA‐A11^R^ hESCs (Figure S8D). These seven potential off‐target genes were confirmed by next generation sequencing (NGS), almost no off‐target probability was observed^30^ (Table S6). Overall, we generated customized immunocompatible hESCs with enhanced safety by installing an effective inducible suicide system. Both engineered hESC lines retained normal pluripotency and karyotypes consistent with their WT counterparts, with almost no risks of off‐target. These results implied that our engineered cells might be safe as therapeutic cells in clinical applications if they could meet a range of quality controls under good manufacturing practice (GMP) conditions.

## 4 DISCUSSION

**FIGURE 6:**
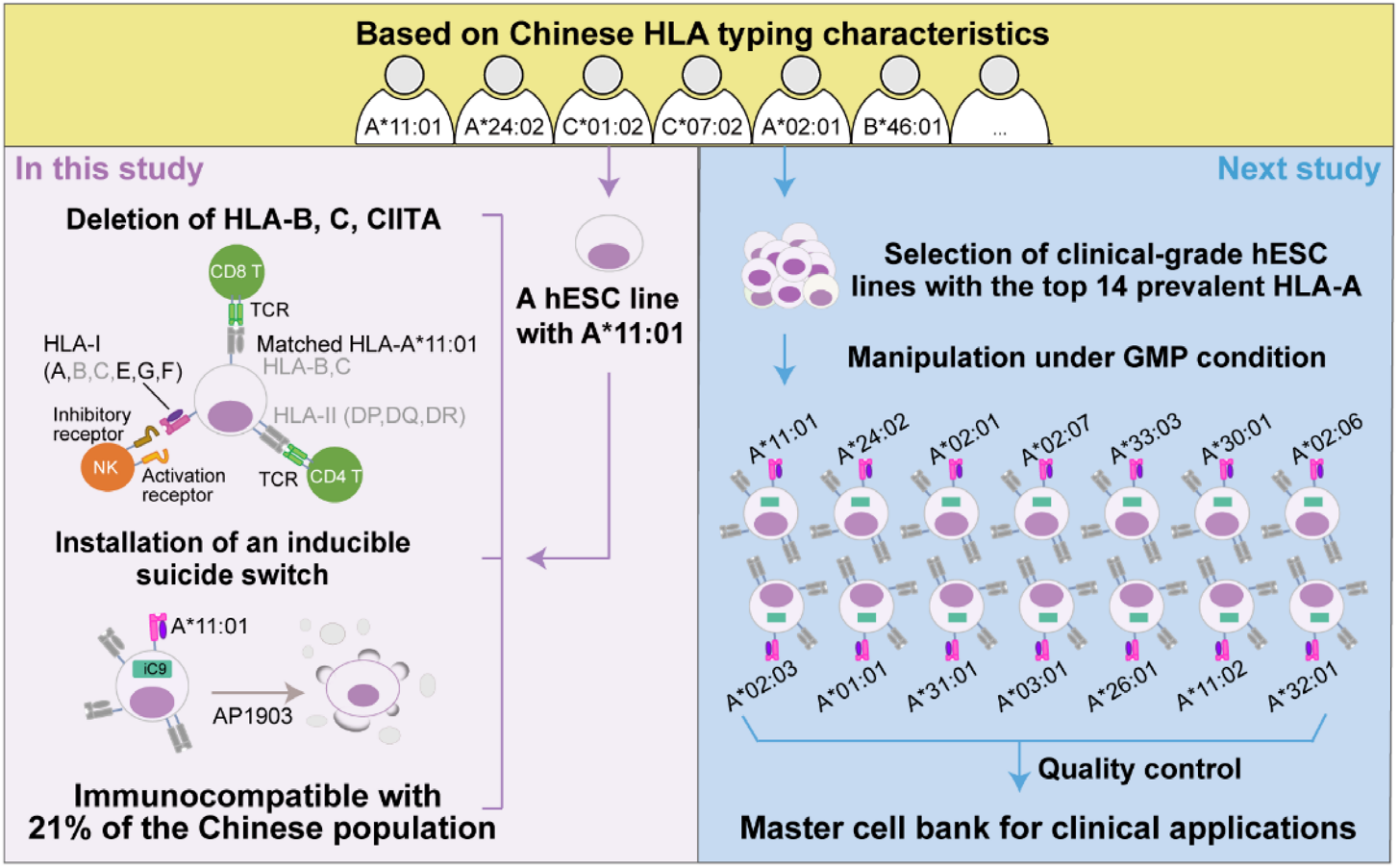
Schematic summary and future perspectives on our human leucocyte antigen (HLA)‐A‐retained strategy. Left: Schematic summary of the customized HLA‐A11‐retained human embryonic stem cell (hESC) line in this study, which is immunocompatible with ~21% of the Chinese population. Right: Future perspectives of our HLA‐A‐retained customized strategy based on Chinese HLA typing. Manipulation process of 14 clinical‐grade hESC lines with high‐frequency HLA‐A under good manufacturing practice (GMP) conditions could enable establishment of a wide‐coverage stem cell bank involving 14 HLA‐A‐retained hESC lines, which covers ~95% of the Chinese population and other broad populations worldwide.

In this study, we have generated an individualized immunocompatible hESC line with safety guarantee using the HLA‐A*11:01‐retained strategy, which covers roughly 21% of the Chinese population (Figure 6). According to data showing that 95% of the Chinese population could be covered by 14 HLA‐A^R^ or 37 HLA‐B^R^ or 18 HLA‐C^R^ hESC lines (Figure S1A and Table S7), this HLA‐A‐retention strategy allows more extensive populations to be covered with a minimum number of cell lines. The 14 most prevalent HLA‐A alleles in the Chinese population are A*11:01, A*24:02, A*02:01, A*02:07, A*33:03, A*30:01, A*02:06, A*02:03, A*01:01, A*31:01, A*03:01, A*26:01, A*11:02, and A*32:01. Subsequently, we counted the distribution of these 14 HLA‐A alleles in Australia (65%), Europe (82%), North America (71%), North‐East Asia (86%), North African (49%), Oceania (98%), South Asia (75%), South‐Central America (60%), South‐East Asia (89%), Sub‐Saharan Africa (46%), and Western Asia (51%; Figure S1D and Table S9). Next, based on compliant hESC resources established by the National Stem Cell Resource Bank and the cell preparation platform that complies with GMP regulations, we aim to manipulate the same process on clinical‐grade hESC lines with high‐frequency HLA‐A alleles under GMP conditions. In this way, we could achieve establishment of an HLA‐A^R^ bank of 14 hESC lines covering 95% of the Chinese population and other broad populations worldwide (Figure 6). Establishment of such a universal stem cell bank is essential to support the development of allogeneic hESC‐based therapy. The approach will reduce the cost of preparation but also enable advanced preparation for rapid transplantation for patients with acute diseases. Similarly, Yamanaka and colleagues constructed a clinical‐grade haplobank of 27 iPSC lines from seven donors in accordance with GMP regulations, covering approximately 40% of the Japanese population.^31^ In contrast to haplobanks of iPSCs, which requires multiple strains of cells to cover a broader population, our anticipated HLA‐A^R^ bank of hESCs could be achieved more readily using fewer cell lines.

To generate individualized immunocompatible donor cells, Li and colleagues knocked out *B2M* and transferred the exogenous HLA‐A*11:01 gene to 70% iPSCs by lentiviral infection.^18^ The potential risks associated with this strategy have been considered in our work, including retention of non‐classical HLA‐I, deletion of HLA‐II, and a better approach for integration. We chose Homology directed repair (HDR)‐mediated site‐specific integration to knockin our genes of interest, promoted by the CAG promoter. Interestingly, we observed that the exogenous genes promoted by the EF1α promoter were silenced in HLA‐A11^R^ hESCs (data not shown) in accordance with the results generated by Carman and co‐workers.^32^ Further studies are required to compare the expression of integrated genes promoted by different promoters in hPSCs.

Besides incompatibility with the immune system, hPSC‐based therapy also faces the challenge of tumorigenicity, which can be caused by undifferentiated and/or immature cells, genetic mutations,^33^ or infections. The presentation of tumour antigens by HLA‐I is critical to the success of clearance aimed at stimulating anti‐tumour CD8^+^ T‐cell responses.^13^, ^34^ HLA‐A11^R^ hESCs retained the capacity to present internal ‘danger signals’ by the HLA‐A11 molecule with immune surveillance. Immune surveillance should acquire more attention in stem cell‐based applications, whereas cancer cells are transmissible in other species (e.g., Tasmanian devil).^35^, ^36^

To further ensure safety, the suicide gene approach is an additional safeguard. We installed a drug‐inducible suicide system into HLA‐A11^R^ hESCs. We showed that iC9‐HLA‐A11^R^ hESCs could be induced to commit suicide efficiently in vitro by AP1903, used in clinical trials.^37^, ^38^, ^39^ Although the drug‐inducible suicide system was not conducted in a mouse model here, previous studies have indicated that this system is effective in vivo.^32^, ^40^ Suicide switches may reduce risks, but also abrogate the potential for functional effects. Hence, a drug‐regulatable system may provide a more attractive safety guarantee for functional effects without ablating the transplanted cells permanently.^41^

In general, we generated a pilot immunocompatible hESC line with safety insurance that could be used to cover ~21% of the Chinese population. These hypoimmunogenic hESCs can be protected from allogeneic immune cells while also maintaining immune surveillance competence. We aim to establish an HLA‐A^R^ bank of 14 hESC lines as a source of next‐generation donor cells for regenerative medicine to cover 95% of the Chinese population and other broad populations around the world.

## AUTHOR CONTRIBUTIONS

Qi Zhou and Zheng Hu conceived this study. Tian‐Tian Ji, Shuai‐Shuai Niu, and Ming‐Hui Fang designed the experiments. Tian‐Tian Ji, Shuai‐Shuai Niu, Ming‐Hui Fang, Ling‐Xue Xu, Jun Zou, Fei Xu, Meng Zhang, Rui Niu, and Lei Wang performed the experiments. Xin Wang performed the bioinformatic analysis. Tian‐Tian Ji, Shuai‐Shuai Niu, and Ming‐Hui Fang analysed the data and wrote the article. Qi Zhou, Zheng Hu, Wei Li, Jie Hao, Jun Wu, Zhi‐Kun Li, and Yong‐Guang Yang reviewed and edited the article. All the authors have read and approved the article.

## FUNDING INFORMATION

This work was supported by the National Key Research and Development Program (2018YFA0108400, 2019YFA0110900, 2019YFA0903800, 2021YFA1101600, 2020YFA080400, 2021YFA1100701), Strategic Priority Research Program of Chinese Academy of Sciences (XDA16021102, XDA16030701, XDA16030702, XDA16040502, XDA16040504), the National Natural Science Foundation of China (82241224), and the International Cooperation Project of China Manned Space Program.

## CONFLICT OF INTEREST STATEMENT

All authors declare that they have no competing interests. The study about hESCs was performed according to the guidelines of the Animal and Medical Ethics Committee of the Institute of Zoology, Chinese Academy of Sciences. All animal experiments were performed according to the guidelines of the Institutional Animal Care and Use Committee of the First Hospital of Jilin University. Construction of humanized mice by discarded human tissues was approved by the Institutional Review Board of the first hospital of Jilin University.

## Supporting information


**FIGURE S1:** Percentage coverage by human leucocyte antigen (HLA) in different populations. (A) HLA‐A, HLA‐B, and HLA‐C allele frequencies among the 169,995 donors in the CMDP registry in China. Fourteen strains of HLA‐A^R^, 37 strains of HLA‐B^R^, or 18 strains of HLA‐C^R^ human embryonic stem cell (hESC) are required to cover >95% of the population in China. (B) The top‐14 HLA‐A allele frequencies in eight regions of China. (C) Allele frequency of HLA‐A*11:01 in these areas in the world. (D) Allele frequency of the Chinese top‐14 most prevalent HLA‐A alleles in these areas around the world.
**FIGURE S2:** Generation of human leucocyte antigen (HLA)‐A11^R^ human embryonic stem cells (hESCs). (A) Agarose gel electrophoresis for PCR‐genotyping products for engineered HLA‐A11^R^ hESCs. Red arrowheads indicate selected clones for the subsequent experiment (HLA‐A, B^R^#1, HLA‐A11^R^ #32). Blue arrowheads indicated the wild‐type (WT) band. (B) PCR confirmation of knockout of HLA‐B, HLA‐C, and CIITA is related to Figure 1B. (C) Sanger sequencing reveals that in the HLA‐A11^R^ cell line, 3312 bp were deleted on both HLA‐B alleles, 4633 bp were deleted on both HLA‐C alleles, 71 bp were deleted on one CIITA allele and 18 (8 + 10) bp were deleted from the other CIITA allele.
**FIGURE S3:** Differentiation of human embryonic stem cells (hESCs) to endothelial cells. (A) Schematic illustration of the endothelial cell (EC) differentiation strategy for hESCs. (B) Typical phases of EC differentiation were shown by phase‐contrast imaging. hESCs stage on Day 1, mesoderm stage on Day 4, and EC stage on Day 6. Scale bars, 200 μm for Day 1. 10 and 100 μm for Days 4 and 6. (C) Representative flow cytometry assay for CD31 and CD144 expression on hESC‐ECs differentiated from wild‐type (WT) or human leucocyte antigen (HLA)‐A11^R^ hESCs. (D) Histograms displaying the differentiation efficiency of hESC‐ECs from WT or HLA‐A11^R^ hESCs. Columns show the mean ± SD of three independent experiments. ECs were counted by CD144 and CD31 double positive staining. Isotype staining was used as the negative control.
**FIGURE S4:** Pluripotent characterization of engineered human embryonic stem cells (hESCs). (A) Relative expression of pluripotency transcription factors (SOX2, OCT4) was measured by real‐time quantitative PCR in WT, HLA‐A11^R^, and iC9‐HLA‐A11^R^ hESCs. (B) Expression of pluripotency markers (SSEA4, TRA‐1‐60, TRA‐1‐81) at the membrane surface was measured by flow cytometry analysis in wild‐type (WT), human leucocyte antigen (HLA)‐A11^R^, and iC9‐HLA‐A11^R^ hESCs. (C) Immunofluorescence staining for the hESC markers OCT4, SOX2, SSEA4, and NANOG of iC9‐HLA‐A11^R^ hESCs. DNA was stained with Hoechst. Scale bars, 48.3 μm. (D) Pluripotency was confirmed by the formation of WT or HLA‐A11^R^‐derived teratomas containing tissues from all three germ layers (ectoderm, mesoderm, and endoderm). Scale bars, 50 μm.
**FIGURE S5:** Flow cytometry (FCM) data on T‐cell responses and NK cell degranulation. (A) T‐cell proliferation was detected by the CFSE assay: the percentages of proliferative CD3^+^ (top panel), CD4^+^ (middle panel), and CD8^+^ (bottom panel) T cells were plotted by gating of the reduced CFSE fluorescence. T cells cultured alone were used as Neg. Ctrl; T cells treated with CD3/CD28 beads served as Pos. Ctrl. (B,C) FCM for the expression of T‐cell activation marker CD69 (left) and CD154 (right): wild‐type (WT) or human leucocyte antigen (HLA)‐A11^R^ ECs were co‐cultured with T cells for 3 days (B) and 6 days (C) from one representative donor. Pos. Ctrl and Neg. Ctrl were the same as (A). (D) FACS contour plots of NK cell degranulation assay from one representative donor. CD107a^+^ cells served as a readout for NK cell degranulation against the stimulation of WT or HLA‐A11^R^ ECs. NK cells cultured alone were used as Neg. Ctrl. NK cells treated with PMA/ionomycin were set as Pos. Ctrl.
**FIGURE S6:** The phenotype of humanized mice. (A) The percentage of human CD45^+^, CD19^+^, CD3^+^, CD4^+^, CD8^+^, CD33^+^, and CD56^+^ cells in PBMCs and spleen at Week 8 after hESCs injection. (B) The percentage of CD45RO^+^ and CD45RA^+^ cells in T cells (CD3^+^, CD4^+^, and CD8^+^ cells) of PBMCs and spleen at Week 8 after hESCs injection. (C) The ratios of distinct subsets of human immune cells in PBMCs of humanized mice before ECs transplantation. (D) Area size statistics for wild‐type (WT) and human leucocyte antigen (HLA)‐A11^R^ EC‐formed lumps in humanized mice (collected at week 3).
**FIGURE S7:** Generation of iC9‐human leucocyte antigen (HLA)‐A11^R^ cell lines and confirmation of the AP1903‐inducible apoptosis system of iC9‐HLA‐A11^R^ human embryonic stem cells (hESCs) *in vitro*. (A) Agarose gel electrophoresis for PCR‐genotyping products for engineered iC9‐HLA‐A11^R^ hESCs, only the knockin sample has a band. Red arrowheads indicate the selected clone for the following experiment (iC9‐HLA‐A11^R^#9). (B) PCR confirmation of iC9 knockin related to Figure 5A. (C) Cells were cultured for 24 or 8 h in 96‐well culture plates in the presence of AP1903 with gradient concentration. (D) Cell proliferation results for wild‐type (WT), human leucocyte antigen (HLA)‐A11^R^, and iC9‐HLA‐A11^R^ hESCs. (E) Relative high expression of iC9 in iC9‐HLA‐A11^R^ hESCs compared with that in WT and HLA‐A11^R^ hESCs. (F) Flow cytomentry analysis of iC9‐HLA‐A11^R^ hESC apoptosis (annexin V) upon AP1903 (10 nM) treatment at different time points.
**FIGURE S8:** WGS‐based off‐target analysis of engineered cell lines (A) Flow chart of the analytical pipeline for WGS. (B) Potential off‐target sites of each gRNAs identified by Cas‐OFFinder in the hg38 human genome. The PAM sequence contains NAG, NGA, and NGG. (C) Summary of potential off‐target information for human leucocyte antigen (HLA)‐A11^R^ and iC9‐HLA‐A11^R^ human embryonic stem cells (hESCs) by WGS data analysis dependent on gRNAs. The parental non‐edited cell line (WT hESCs) was used as a reference control. (D) Summary of potential off‐target information for HLA‐A11^R^ and iC9‐HLA‐A11^R^ hESCs by gRNA‐independent analysis of WGS, but related to cancer genes. The WT hESC line was used as a reference control.
Supplementary methods
**TABLE S1:** CRISPR gRNA Sequences and editing efficiency
**TABLE S2:** Primers used in genotyping, qRT‐PCR, gRNA selection, and pre‐index PCR for NGS
**TABLE S3:** Genomic indel patterns of the genome‐edited clones
**TABLE S4:** Karyotype analysis statistics of wild‐type (WT), human leucocyte antigen (HLA)‐A11^R^, and iC9‐HLA‐A11^R^ human embryonic stem cells (hESCs)
**TABLE S5:** Human leucocyte antigen (HLA) haplotype information of human embryonic stem cells (hESCs), PBMCs, and hu‐mice used in this study
**TABLE S6:** Summary of potential off‐target information for human leucocyte antigen (HLA)‐A11^R^ human embryonic stem cells (hESCs) by WGS and confirmed by NGS
**TABLE S7:** Coverage percentage by human leucocyte antigen (HLA)‐A, HLA‐B, and HLA‐C in the Chinese population
**TABLE S8:** Top 20 human leucocyte antigen (HLA)‐A allele frequencies by geographical region in China
**TABLE S9:** Summary of 14 human leucocyte antigen (HLA)‐A alleles in each geographical region in the world

## Data Availability

All experimental methods, code, software, data, and study materials are available to other researchers upon reasonable request.
