# Supplementary material for: Genome editing HLA alleles for a pilot immunocompatible hESC line in a Chinese hESC bank for cell therapies
Source: Cell Prolif. 2023 May 17;56(5):e13471. doi: 10.1111/cpr.13471 (PMC10212709; doi:10.1111/cpr.13471)
Supplement: Supplementary file 1 — FIGURE S1: Percentage coverage by human leucocyte antigen (HLA) in different populations. (A) HLA‐A, HLA‐B, and HLA‐C allele frequencies among the 169,995 donors in the CMDP registry in China. Fourteen strains of HLA‐AR, 37 strains of HLA‐BR, or 18 strains of HLA‐CR human embryonic stem cell (hESC) are required to cover >95% of the population in China. (B) The top‐14 HLA‐A allele frequencies in eight regions of China. (C) Allele frequency of HLA‐A*11:01 in these areas in the world. (D) Allele frequency of the Chinese top‐14 most prevalent HLA‐A alleles in these areas around the world. FIGURE S2: Generation of human leucocyte antigen (HLA)‐A11R human embryonic stem cells (hESCs). (A) Agarose gel electrophoresis for PCR‐genotyping products for engineered HLA‐A11R hESCs. Red arrowheads indicate selected clones for the subsequent experiment (HLA‐A, BR#1, HLA‐A11R #32). Blue arrowheads indicated the wild‐type (WT) band. (B) PCR confirmation of knockout of HLA‐B, HLA‐C, and CIITA is related to Figure 1B. (C) Sanger sequencing reveals that in the HLA‐A11R cell line, 3312 bp were deleted on both HLA‐B alleles, 4633 bp were deleted on both HLA‐C alleles, 71 bp were deleted on one CIITA allele and 18 (8 + 10) bp were deleted from the other CIITA allele. FIGURE S3: Differentiation of human embryonic stem cells (hESCs) to endothelial cells. (A) Schematic illustration of the endothelial cell (EC) differentiation strategy for hESCs. (B) Typical phases of EC differentiation were shown by phase‐contrast imaging. hESCs stage on Day 1, mesoderm stage on Day 4, and EC stage on Day 6. Scale bars, 200 μm for Day 1. 10 and 100 μm for Days 4 and 6. (C) Representative flow cytometry assay for CD31 and CD144 expression on hESC‐ECs differentiated from wild‐type (WT) or human leucocyte antigen (HLA)‐A11R hESCs. (D) Histograms displaying the differentiation efficiency of hESC‐ECs from WT or HLA‐A11R hESCs. Columns show the mean ± SD of three independent experiments. ECs were counted by CD144 a [file CPR-56-e13471-s001.docx]

Supplementary Materials for

Genome editing HLA alleles for a pilot immunocompatible hESC line in a Chinese hESC bank for cell therapies

**This PDF file includes:**

Fig S1 to S8

Supplementary methods

Captions for Table S1 to S9

**
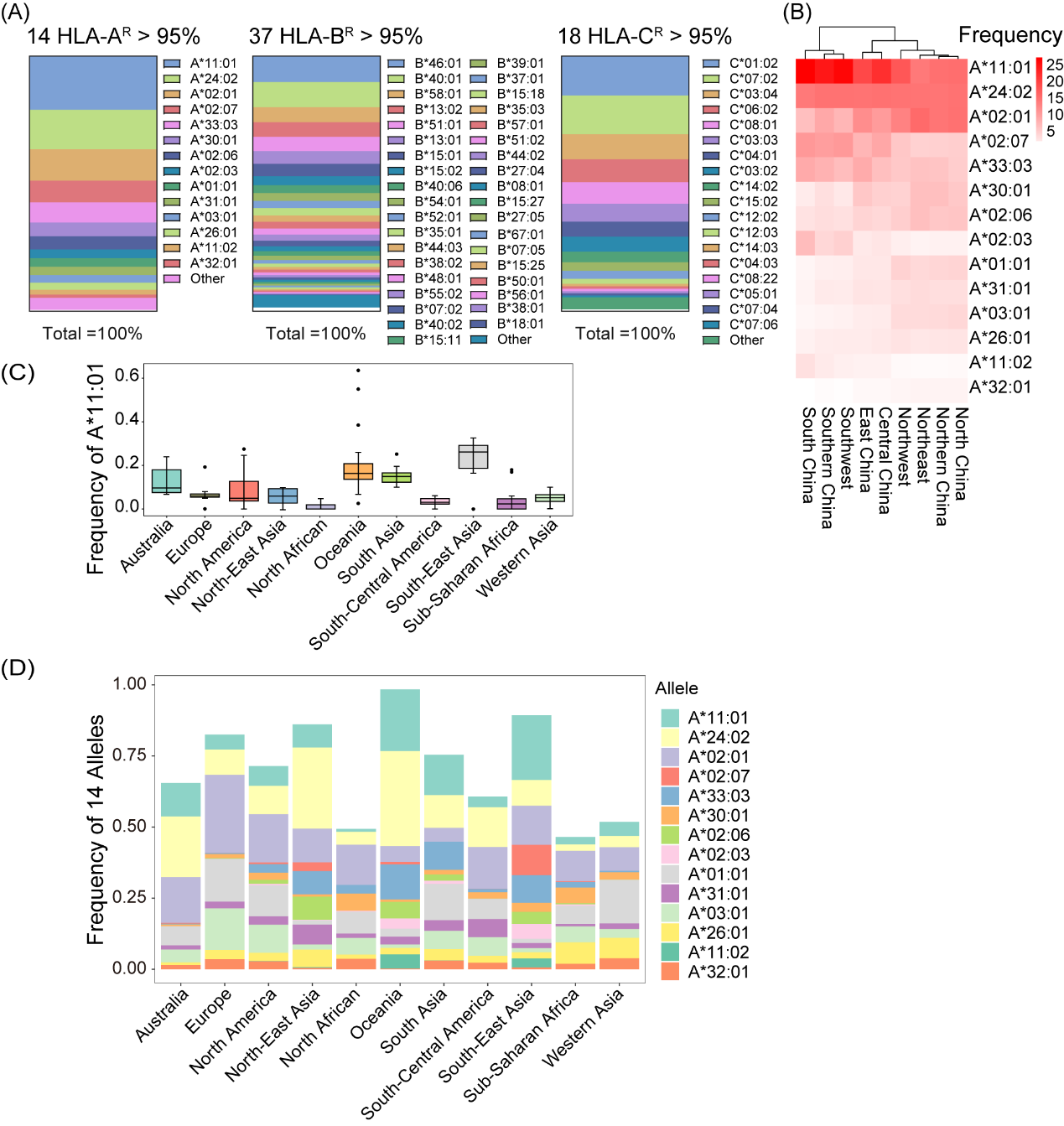
**

**Figure S1. Percentage coverage by HLA in different populations.** (A) HLA-A, -B, and -C allele frequencies among the 169,995 donors in the CMDP registry in China. Fourteen strains of HLA-A^R^, 37 strains of HLA-B^R^, or 18 strains of HLA-C^R^ hESC are required to cover >95% of the population in China. (B) The top-14 HLA-A allele frequencies in eight regions of China. (C) Allele frequency of HLA-A*11:01 in these areas in the world. (D) Allele frequency of the Chinese top-14 most prevalent HLA-A alleles in these areas around the world.

**
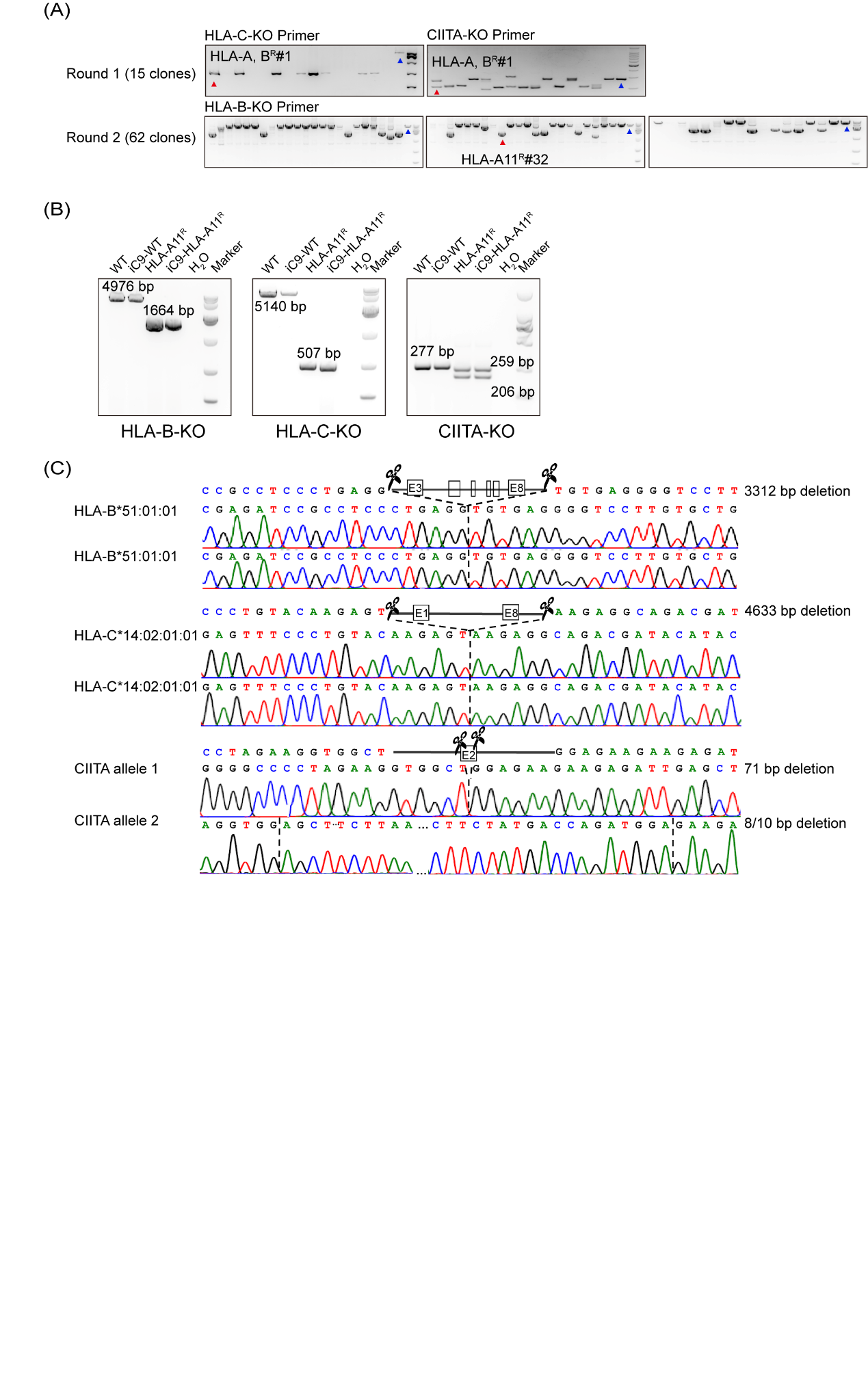
**

**Figure S2. Generation of HLA-A11^R^ hESCs.** (A) Agarose gel electrophoresis for PCR-genotyping products for engineered HLA-A11^R^ hESCs. Red arrowheads indicate selected clones for the subsequent experiment (HLA-A, B^R^#1, HLA-A11^R^ #32). Blue arrowheads indicated the WT band. (B) PCR confirmation of knockout of HLA-B, HLA-C, and CIITA is related to Figure 1B. (C) Sanger sequencing reveals that in the HLA-A11^R^ cell line, 3312 bp were deleted on both HLA-B alleles, 4633 bp were deleted on both HLA-C alleles, 71 bp were deleted on one CIITA allele and 18 (8+10) bp were deleted from the other CIITA allele.


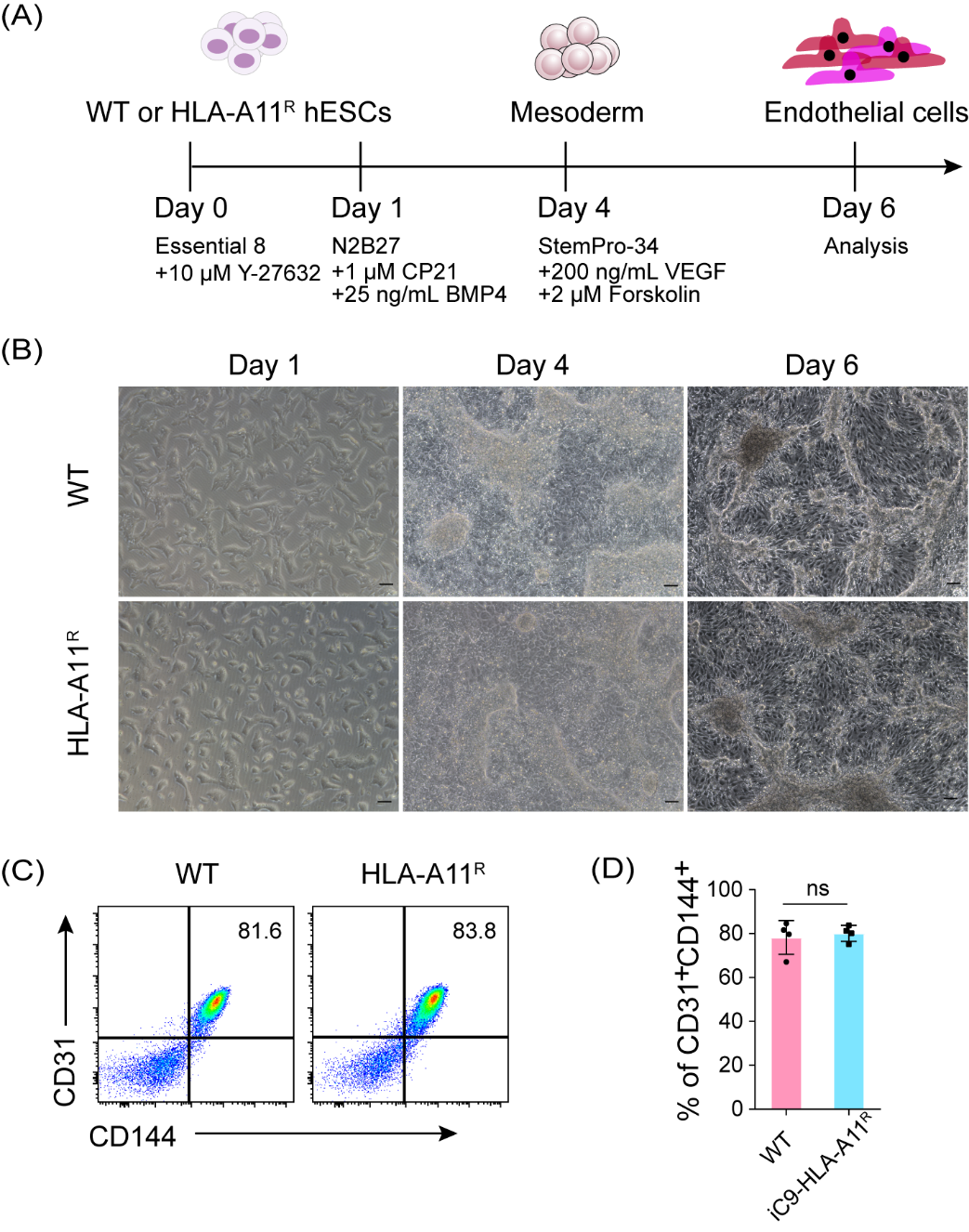


**Figure S3. Differentiation of hESCs to endothelial cells.** (A) Schematic illustration of the EC differentiation strategy for hESCs. (B) Typical phases of EC differentiation were shown by phase-contrast imaging. hESCs stage on Day 1, mesoderm stage on Day 4, and EC stage on Day 6. Scale bars, 200 µm for day 1. 10, 100 µm for day 4 and day 6. (C) Representative FCM assay for CD31 and CD144 expression on hESC-ECs differentiated from WT or HLA-A11^R^ hESCs. (D) Histograms displaying the differentiation efficiency of hESC-ECs from WT or HLA-A11^R^ hESCs. Columns show the mean±SD of three independent experiments. ECs were counted by CD144 and CD31 double positive staining. Isotype staining was used as the negative control.

**
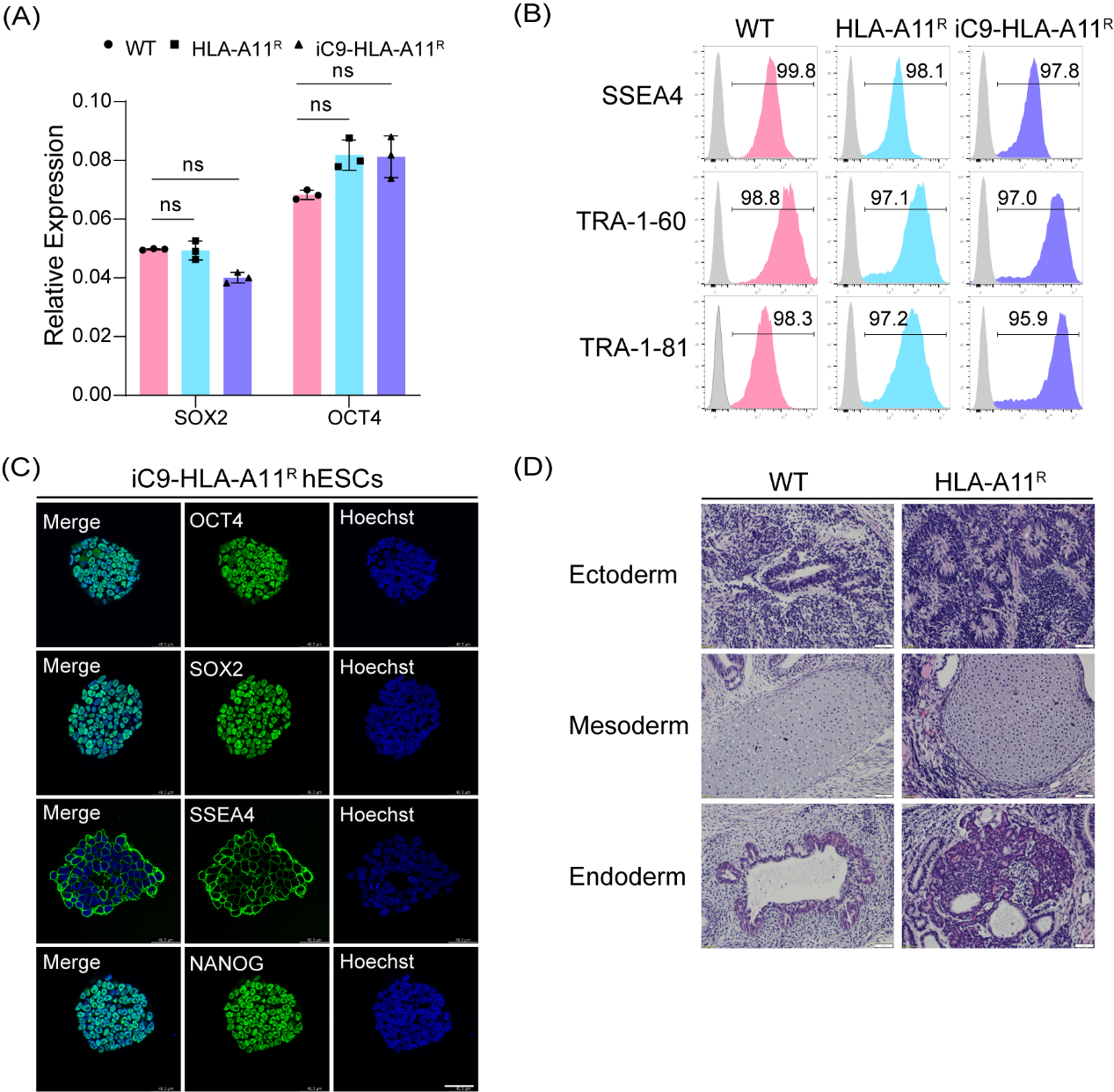
**

**Figure S4. Pluripotent characterization of engineered hESCs.** (A) Relative expression of pluripotency transcription factors (SOX2, OCT4) was measured by real-time quantitative PCR in WT, HLA-A11^R^, and iC9-HLA-A11^R^ hESCs. (B) Expression of pluripotency markers (SSEA4, TRA-1-60, TRA-1-81) at the membrane surface was measured by FCM analysis in WT, HLA-A11^R^, and iC9-HLA-A11^R^ hESCs. (C) Immunofluorescence staining for the hESC markers OCT4, SOX2, SSEA4, and NANOG of iC9-HLA-A11^R^ hESCs. DNA was stained with Hoechst. Scale bars, 48.3 μm. (D) Pluripotency was confirmed by the formation of WT or HLA-A11^R^-derived teratomas containing tissues from all three germ layers (ectoderm, mesoderm, and endoderm). Scale bars, 50 μm.

**
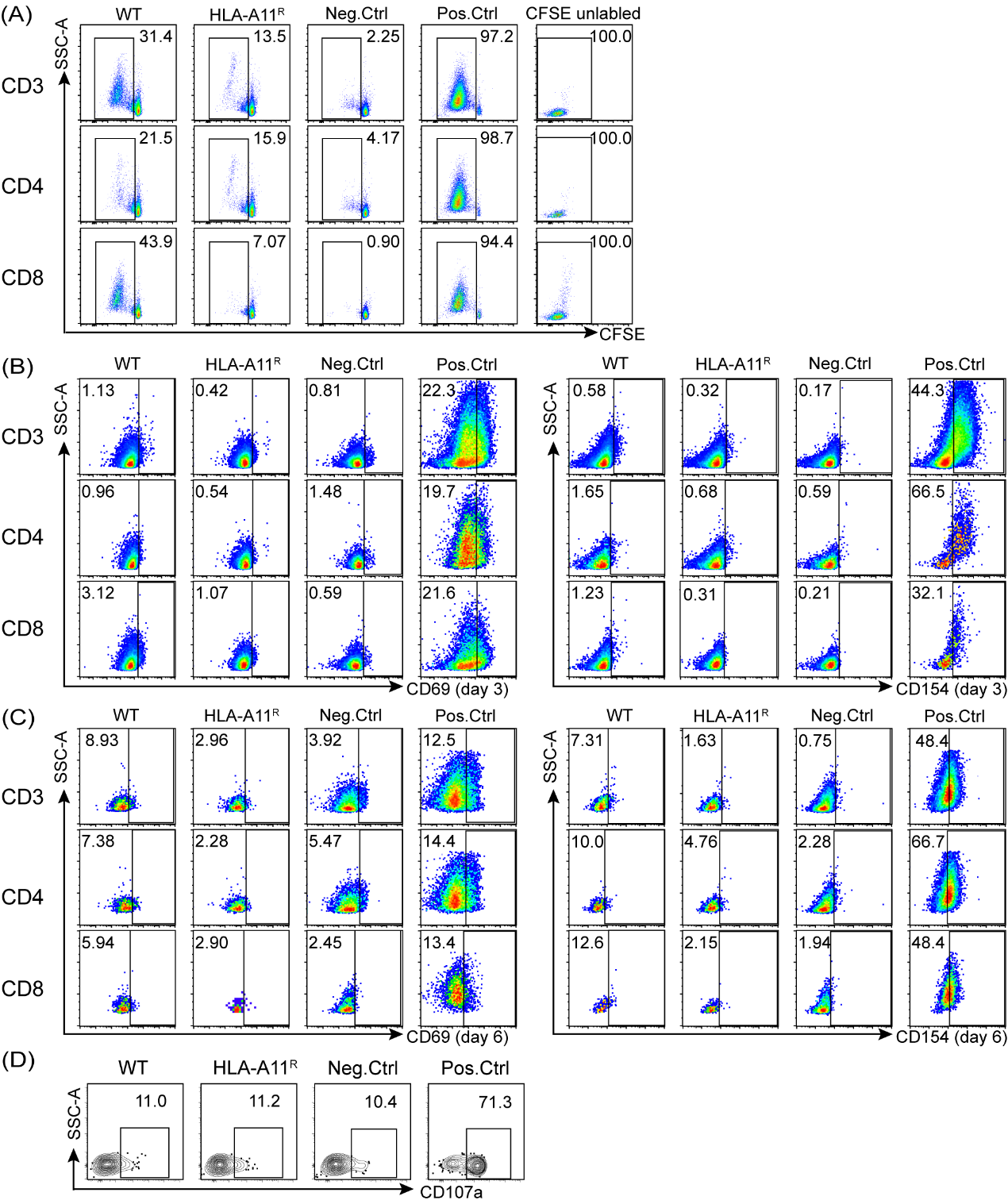
**

**Figure S5. Supplementary FCM data on T cell responses and NK cell degranulation.** (A) T cell proliferation was detected by the CFSE assay: the percentages of proliferative CD3^+^ (top panel), CD4^+^ (middle panel), and CD8^+^ (bottom panel) T cells were plotted by gating of the reduced CFSE fluorescence. T cells cultured alone were used as Neg. Ctrl; T cells treated with CD3/CD28 beads served as Pos. Ctrl. (B-C) FCM for the expression of T cell activation marker CD69 (left) and CD154 (right): WT or HLA-A11^R^ ECs were cocultured with T cells for 3 days (B) and 6 days (C) from one representative donor. Pos. Ctrl and Neg. Ctrl were the same as S5A. (D) FACS contour plots of NK cell degranulation assay from one representative donor. CD107a^+^ cells served as a readout for NK cell degranulation against the stimulation of WT or HLA-A11^R^ ECs. NK cells cultured alone were used as Neg. Ctrl. NK cells treated with PMA/ionomycin were set as Pos. Ctrl.

**
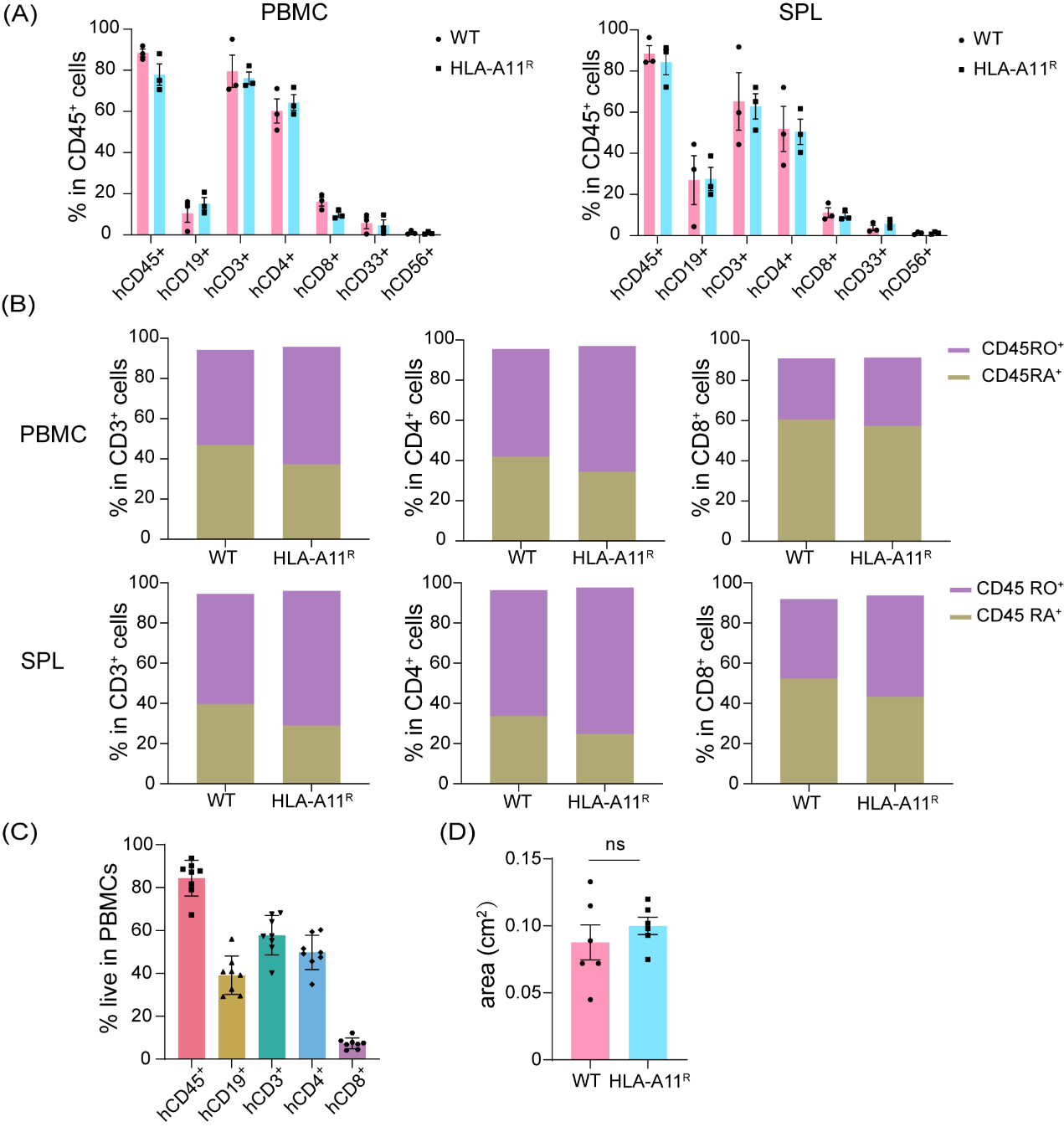
**

**Figure S6.** **The phenotype of humanized mice.** (A) The percentage of human CD45^+^, CD19^+^, CD3^+^, CD4^+^, CD8^+^, CD33^+^, and CD56^+^ cells in PBMCs and spleen at week 8 after hESCs injection. (B) The percentage of CD45RO^+^ and CD45RA^+^ cells in T cells (CD3^+^, CD4^+^, and CD8^+^ cells) of PBMCs and spleen at week 8 after hESCs injection. (C) The ratios of distinct subsets of human immune cells in PBMCs of humanized mice before ECs transplantation. (D) Area size statistics for WT and HLA-A11^R^ EC-formed lumps in humanized mice (collected at week 3).

**
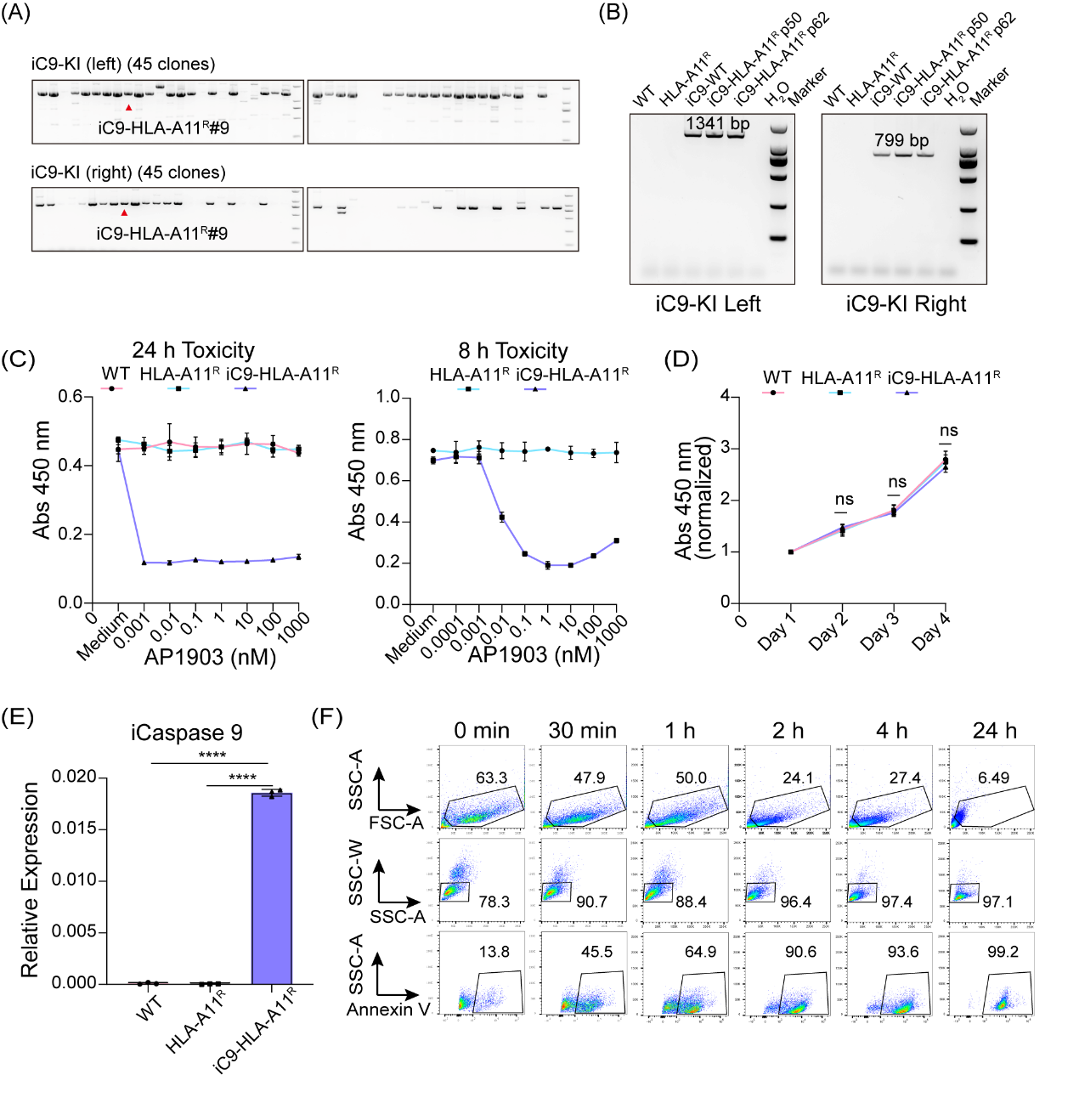
**

**Figure S7. Generation of iC9-HLA-A11^R^ cell lines and confirmation of the AP1903-inducible apoptosis system of iC9-HLA-A11^R^ hESCs *in vitro*.** (A) Agarose gel electrophoresis for PCR-genotyping products for engineered iC9-HLA-A11^R^ hESCs, only the knock-in sample has a band. Red arrowheads indicate the selected clone for the following experiment (iC9-HLA-A11^R^#9). (B) PCR confirmation of iC9 knock-in related to Figure 5A. (C) Cells were cultured for 24 h or 8h in 96-well culture plates in the presence of AP1903 with gradient concentration. (D) Cell proliferation results for WT, HLA-A11^R^, and iC9-HLA-A11^R^ hESCs. (E) Relative high expression of iC9 in iC9-HLA-A11^R^ hESCs compared with that in WT and HLA-A11^R^ hESCs. (F) FCM analysis of iC9-HLA-A11^R^ hESC apoptosis (annexin V) upon AP1903 (10 nM) treatment at different time points.


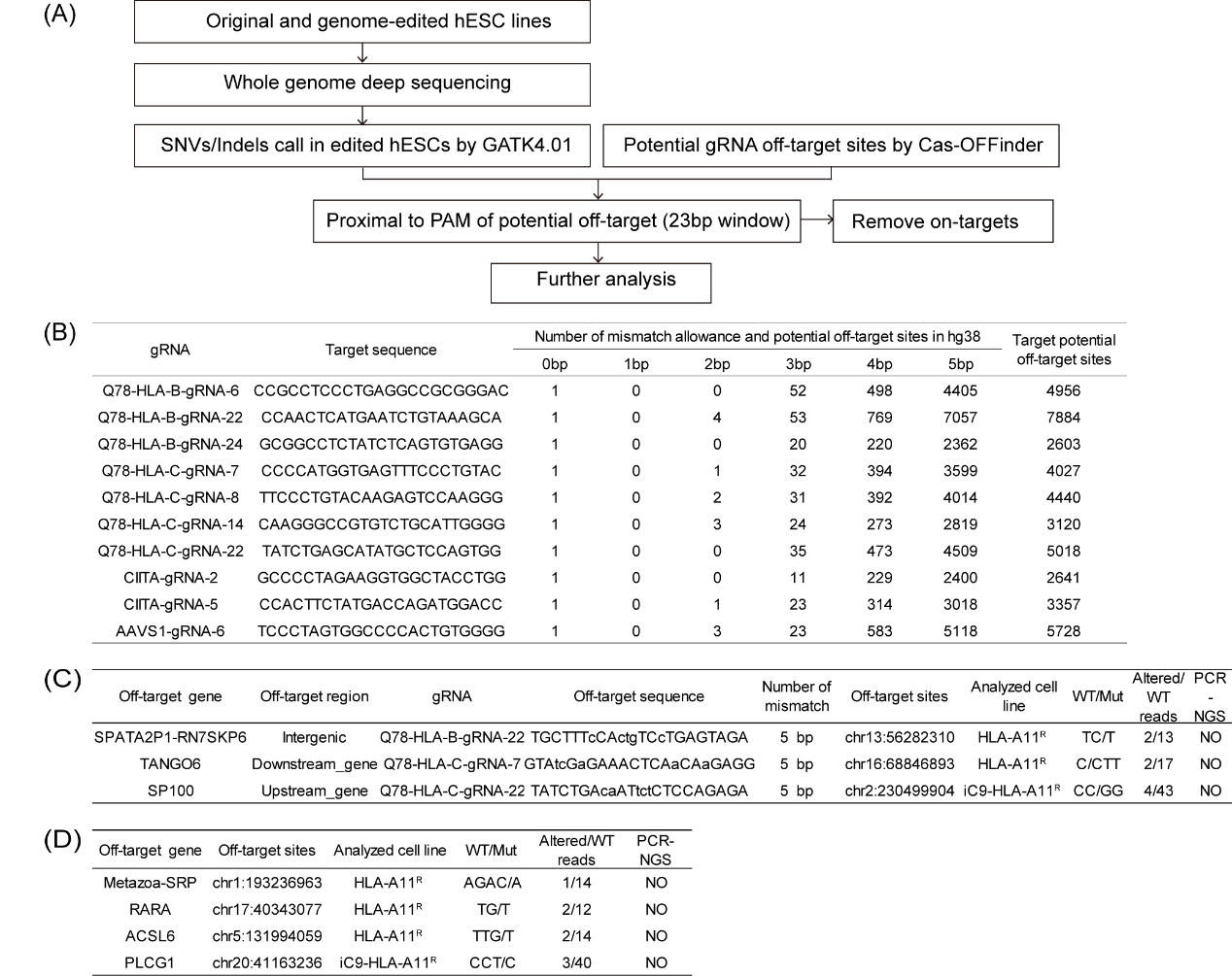


**Figure S8. WGS-based off-target analysis of engineered cell lines** (A) Flow chart of the analytical pipeline for WGS. (B) Potential off-target sites of each gRNAs identified by Cas-OFFinder in the hg38 human genome. The PAM sequence contains NAG, NGA, and NGG. (C) Summary of potential off-target information for HLA-A11^R^ and iC9-HLA-A11^R^ hESCs by WGS data analysis dependent on gRNAs. The parental non-edited cell line (WT hESCs) was used as a reference control. (D) Summary of potential off-target information for HLA-A11^R^ and iC9-HLA-A11^R^ hESCs by gRNA-independent analysis of WGS, but related to cancer genes. The WT hESC line was used as a reference control.

**Supplementary methods**

**Pluripotency analysis by flow cytometry**

The cultured hESCs were detached from plates and suspended in pre-cooled PBS containing 2% FBS, washed twice before being mixed with the following diluted antibodies: PE Mouse anti-Human TRA-1-81 Antigen (BD biosciences, 560885), PE Mouse anti-Human TRA-1-60 Antigen (BD biosciences, 560884), PE Mouse anti-SSEA-4 (BD biosciences, 560128). We typically use 1E6 cells in a 100-µL experimental sample with 1 µL antibody, incubating on ice for 20 minutes in the dark. Cells were then washed twice, centrifuged at 200g for 3 minutes, resuspended, and loaded on FCM (BD LSRFortessa).

**Reverse transcription and quantitative real-time PCR**

RNA was extracted using TRIzol (Invitrogen, 10296028) and the PureLink RNA Mini Kit (Invitrogen, 12183025). The cDNA was synthesized with ReverTra Ace qPCR RT Master Mix with the gDNA remover kit (TOYOBO, FSQ-301) according to the manufacturer’s instructions. Real-time PCR was performed with the THUNDERBIRD SYBR qPCR Master Mix kit (TOYOBO, QPS-201T). All primers used for quantitative real-time PCR are listed in **Table S2**.

**Isolation of T cells and NK cells from PBMC**

Human peripheral blood mononuclear cells used for this study were purchased from Milestone (Shanghai) Biological Science & Technology Co., Ltd (A10Z849104; A10k792066; P121051405C). HLA haplotype information of PBMC was listed in **Table S5**. Quickly thaw the PBMCs in a 37 ℃ water bath and suspended in RPMI 1640 (Gibco) supplemented with 10% human serum (Gemini, 100-512), 1% Penicillin-Streptomycin, 1% GlutaMax (Gibco) and 50 μM 2-beta-mercaptoethanol (full 1640 medium). Then the PBMCs were sorted by FACS for CD3^+^ T cells and CD3^-^CD56^+^ NK cells by using anti-human CD3 (Biolegend, 317318), and anti-human CD56 (Invitrogen, 12056742). Isolated T cells were cultured in full 1640 medium supplemented with 20 U/ml IL-2 (Peprotech, AF-200-02-10). Isolated NK cells were cultured in full 1640 medium.

**Differentiation into endothelial cells**

The protocol of hESC-derived ECs was referenced as described previously. Briefly, 5×10^5^ hESCs were seeded on six-well plates coated with growth-factor-reduced Matrigel in Essential 8 medium. The medium was changed to N2B27 media supplemented with 1 μM CP21 (Selleck, S7954) and 25 ng/ml BMP4 (Peprotech, AF-120-05) on Day 2. After 3 days, the medium was replaced by StemPro-34 (Gibco, 10640-019) supplemented with 200 ng/ml VEGF (Peprotech, AF-100-20) and 2 µM forskolin (Merk, 344270) for 2 days to induce ECs. EC differentiation efficiency was detected by anti-huCD144-PE (Biolegend, 348506) and anti-huCD31-APC (Invitrogen, 17-0319-42) through FCM.

**Cell proliferation assay**

Cell proliferation was evaluated using the Cell Counting Kit-8 (CCK-8) (Sigma, 96992). The cells were seeded in 96-well culture plates, cultured for different periods, or treated with different concentrations of AP1903. Subsequently, E8 medium mixed with 1/10 volume of CCK-8 reagent was added into each well and incubated for 4 hours. The absorbance of each well was measured at 450 nm by the spectrophotometer (PerkinElmer).

**Karyotyping analysis**

The hESCs were treated with 50 ng/mL Colcemid (Invitrogen, 15212012) for six hours. Cells were dissociated and collected by centrifugation. Pellets were resuspended gently by hypotonic solution (0.075 M KCl) at 37°C for 30 minutes, then cells were fixed with pre-cooled fix solution (methanol: glacial acetic acid = 3: 1) on ice for 20 minutes. This step was repeated once. Then pellets were resuspended in 200 μL cold fix solution. 10 μL cell suspension was dropped upon cold glass slides by gravity. Slides were stained with Giemsa staining solution (EKEAR) and observed by a Light microscope. All standard G-banding karyotyping results were analyzed and reported containing numbers, pairing, order, and morphology.

**Whole Genome Sequencing (WGS)**

The genomic DNA was extracted by using E.Z.N.A MicroElute Genomic DNA Kit (Omega) following the manufacturer’s instructions. The quality and concentration of isolated genomic DNA were respectively verified and measured by agarose gel and Qubit DNA Assay Kit in Qubit 2.0 Fluorometer (Life Technologies). The genomic DNA sample was extracted and enzymatically disrupted into fragments, which were ligated with the adapter to form paired-end libraries for Illumina sequencing. The clustering of the index-coded samples was performed on a cBot Cluster Generation System with the NovaSeq 6000 S4 Reagent Kit (Illumina) in accordance with the manufacturer’s recommendations. The DNA libraries were then sequenced on Illumina NovaSeq 6000 platform and 150 bp pair-end reads with at least 30X average sequence depth were generated.

**WGS-based off-target analysis**

The sequenced FASTQ files were mapped to the reference human genome (hg38) by bowtie2 (Ver0.12.9), and duplicated reads were removed by homemade script. Each bam file was analyzed by GATK (Ver41.3.0) to find genome-edited hESC-specific SNVs and indels based on default parameters. We focused on small indels as potential off-target mutations. Using three types of PAM (NGG, NAG, and NGA) for further analysis, we also predicted potential gRNA binding sites with mismatches up to 5 bp using Cas-OFFinder. Identified indels within the off-target sites’ windows were retained for further analysis. Using COSMIC (Ver.85), we further extracted potentially oncogenic mutations located in exonic regions, independent of gRNAs. We confirmed the potential off-target sites by Next-Generation Sequencing (NGS) after index-PCR (**Table S2**).

**Captions for Table S1 to S9**

Table S1. CRISPR gRNA Sequences and editing efficiency

Table S2. Primers used in genotyping, qRT-PCR, gRNA selection, and pre-index PCR for NGS

Table S3. Genomic indel patterns of the genome-edited clones

Table S4. Karyotype analysis statistics of WT, HLA-A11^R^, and iC9-HLA-A11^R^ hESCs

Table S5. HLA haplotype information of hESCs, PBMCs, and hu-mice used in this study

Table S6. Summary of potential off-target information for HLA-A11^R^ hESCs by WGS and confirmed by NGS

Table S7. Coverage percentage by HLA-A, HLA-B, and HLA-C in the Chinese population

Table S8. Top 20 HLA-A allele frequencies by geographical region in China

Table S9. Summary of 14 HLA-A alleles in each geographical region in the world

**Table S1. CRISPR gRNA sequences and editing efficiency**

| **gRNA name** | **gRNA sequence (5’-3’)** | **PAM** | **Indel rate (Sanger sequence)** |
| --- | --- | --- | --- |
| HLA-B-gRNA-6 | CCTCCCTGAGGCCGCGGGAC | -CGG | 74.00% |
| HLA-B-gRNA-22 | ACTCATGAATCTGTAAAGCA | -TGG | 50.00% |
| HLA-B-gRNA-24 | GCGGCCTCTATCTCAGTGTG | -AGG | 100.00% |
| HLA-C-gRNA-7 | CATGGTGAGTTTCCCTGTAC | -GGG | 40.00% |
| HLA-C-gRNA-8 | TTCCCTGTACAAGAGTCCAA | -GGG | 100.00% |
| HLA-C-gRNA-14 | CAAGGGCCGTGTCTGCATTG | -GGG | 58.33% |
| HLA-C-gRNA-22 | TATCTGAGCATATGCTCCAG | -TGG | 100.00% |
| CIITA-gRNA-2 | GCCCCTAGAAGGTGGCTACC | -TGG | 76.92% |
| CIITA-gRNA-5 | CTTCTATGACCAGATGGACC | -TGG | 100.00% |
| AAVS1-gRNA-6 | TCCCTAGTGGCCCCACTGTG | -GGG | 71.40% |

**Table S2. Primers used in genotyping, qRT-PCR, gRNA selection, and pre-index PCR for NGS**

| **Name** | **Primers** | **Sequence (5’-3’)** | **Usage** |
| --- | --- | --- | --- |
| HLA-B-KO PCR | Forward | gcggtcccagttctaaagtccc | PCR |
|  | Reverse | tgccttcctagtagacatctcctcc |  |
| HLA-C-KO PCR | Forward | gcaacaacccagagtcacagaac |  |
|  | Reverse | cagacatcctcatatccctcaaatc |  |
| CIITA-KO PCR | Forward | ttctgcctctttccaacaccc |  |
|  | Reverse | tttcccctgattgccgtctc |  |
| iC9-KI (left) PCR | Forward | ccccacagttggaggagaatcca |  |
|  | Reverse | agggtcgctaatgctgtttcggtg |  |
| iC9-KI (right) PCR | Forward | taatcaatgtcaagctcggggacacaggat |  |
|  | Reverse | accactttgagctctactggcttc |  |
| OCT4 qPCR | Forward | gacagggggaggggaggagctagg | qRT-PCR |
|  | Reverse | cttccctccaaccagttgccccaaac |  |
| Sox2 qPCR | Forward | gggaaatgggaggggtgcaaaagagg |  |
|  | Reverse | ttgcgtgagtgtggatgggattggtg |  |
| Caspase-9 qPCR | Forward | ccacatgccactctcgtctt |  |
|  | Reverse | ggactcacggcagaagttca |  |
| GAPDH qPCR | Forward | gcaccgtcaaggctgagaac |  |
|  | Reverse | atggtggtgaagacgccagt |  |
| AAVS1-gRNA-6-F1 | Forward | ggatcagtgaaacgcaccagaca | PCR |
| AAVS1-gRNA-6-R1 | Reverse | ggatcagtgaaacgcaccagaca |  |
| CIITA-gRNA2, 5-F1 | Forward | caaatcctggttccacttggacatg |  |
| CIITA-gRNA2, 5-R1 | Reverse | ctcaggtgcttcctcaccgatattg |  |
| HLA-B-gRNA6-F7 | Forward | ctccgagagccttgtctgcatt |  |
| HLA-B-gRNA6-R7 | Reverse | ccttcccgttctccaggtgtct |  |
| HLA-B-gRNA22, 24-F6 | Forward | ctgagaaccttccagaatctgcatg |  |
| HLA-B-gRNA22, 24-R6 | Reverse | atctatcgtctgcctcttcccactg |  |
| HLA-C-gRNA7, 8-F6 | Forward | aggggcagagagaagggc |  |
| HLA-C-gRNA7, 8-R6 | Reverse | actgcggagacgctgattgg |  |
| HLA-C-gRNA22-F6 | Forward | tgctgactccaatcccaact |  |
| HLA-C-gRNA22-R6 | Reverse | agacatcctcatatccctcaaatc |  |
| HLA-C-preindex-F1 | Forward primer | acactctttccctacacgacgctcttccgatctactctacacgtccattcccagggcga | Pre-Index PCR for NGS |
| HLA-C-preindex-R1 | Reverse primer | gtgactggagttcagacgtgtgctcttccgatcttgcctcatggcacttaccaccttcc |  |
| HLA-B-preindex-F1 | Forward primer | acactctttccctacacgacgctcttccgatctatcgcgctccgctactacaaccaga |  |
| HLA-B-preindex-R1 | Reverse primer | gtgactggagttcagacgtgtgctcttccgatctgaatgaatgatcactgtagagcacc |  |

**Continued Table S2**

| **Name** | **Primers** | **Sequence (5’-3’)** | **Usage** |
| --- | --- | --- | --- |
| CIITA-preindex-F2 | Forward primer | acactctttccctacacgacgctcttccgatctttctgcctctttccaacaccc | Pre-Index PCR for NGS |
| CIITA-preindex-R2 | Reverse primer | gtgactggagttcagacgtgtgctcttccgatcttttcccctgattgccgtctc |  |
| ACSL6-preindex-F1 | Forward primer | acactctttccctacacgacgctcttccgatctagatcctgaggatactgcgactgc |  |
| ACSL6-preindex-R1 | Reverse primer | gtgactggagttcagacgtgtgctcttccgatctcagtggaacgtctcctatcctgc |  |
| Metazoa-SRP-preindex-F3 | Forward primer | acactctttccctacacgacgctcttccgatctttcagcctgggcaacaagagc |  |
| Metazoa-SRP-preindex-R3 | Reverse primer | gtgactggagttcagacgtgtgctcttccgatcttactcaggaggttgaggcaggag |  |
| PLCG1-preindex-F1 | Forward primer | acactctttccctacacgacgctcttccgatctaccatactagctagcttaccttctct |  |
| PLCG1-preindex-R1 | Reverse primer | gtgactggagttcagacgtgtgctcttccgatctaggaagctgagcatgaactcctg |  |
| RARA-preindex-F1 | Forward primer | acactctttccctacacgacgctcttccgatcttgcccttgctggacaattgaacc |  |
| RARA-preindex-R1 | Reverse primer | gtgactggagttcagacgtgtgctcttccgatctagctgttggattccaggcaagaag |  |
| SPATA2P1-RN7SKP6-preindex-F1 | Forward primer | acactctttccctacacgacgctcttccgatctcagcagaatgggcatactacaagtgg |  |
| SPATA2P1-RN7SKP6-preindex-R1 | Reverse primer | gtgactggagttcagacgtgtgctcttccgatcttgtatgtgtctagtccatacaatc |  |
| SP100-preindex-F2 | Forward primer | acactctttccctacacgacgctcttccgatcttttacacttctcagaggtgagtctga |  |
| SP100-preindex-R2 | Reverse primer | gtgactggagttcagacgtgtgctcttccgatctgcaagatgccagtctatctgcaaag |  |
| TANGO6-preindex-F2 | Forward primer | acactctttccctacacgacgctcttccgatctattacagatgtgagccaccacgtc |  |
| TANGO6-preindex-R2 | Reverse primer | gtgactggagttcagacgtgtgctcttccgatctacgccactgcattccatcctg |  |

**Table S3. Genomic indel patterns of the genome-edited clones**

| **Target cell** | **WT-hESC** | | | |
| --- | --- | --- | --- | --- |
| **Used gRNA(s)** | **HLA-C-gRNA-7, 8, 14, 22. CIITA-gRNA-2, 5** | | | |
| **Sorting** | **HLA-C (-) CIITA (-)** | | | |
| **Allele** | **Clone ID** | **HLA-C** | **CIITA** |  |
|  | **HLA-A,B^R^#1** | **-4633 bp; -4633 bp** | **-71 bp; -8/10 bp** | **Uesd in study** |
|  | HLA-A,B^R^#2 |  | -71 bp |  |
|  | HLA-A,B^R^#3 | -4645 bp; -4645 bp | -62 bp |  |
|  | HLA-A,B^R^#4 |  |  |  |
|  | HLA-A,B^R^#5 |  | -86 bp; WT |  |
|  | HLA-A,B^R^#6 | -4624 bp; -4624 bp | -71 bp |  |
|  | HLA-A,B^R^#7 |  | +1/-65 bp, WT |  |
|  | HLA-A,B^R^#8 |  | -71 bp |  |
|  | HLA-A,B^R^#9 | -4634 bp; -4634 bp | -75bp |  |
|  | HLA-A,B^R^#10 | -4630 bp; -4630 bp |  |  |
|  | HLA-A,B^R^#11 |  | +1/-63 bp |  |
|  | HLA-A,B^R^#12 |  |  |  |
|  | HLA-A,B^R^#13 | -4624 bp; -4624 bp | +1/-63 bp |  |
|  | HLA-A,B^R^#14 | -4625 bp; -4625 bp | -75 bp |  |
|  | HLA-A,B^R^#15 |  |  |  |
|  | Efficiency |  |  | 40% |

**Continued Table S3**

| **Target cell** | **HLA-A,B^R^#1** | | | | |
| --- | --- | --- | --- | --- | --- |
| **Used gRNA(s)** | **HLA-B-gRNA-6, 22, 24** | | | | |
| **Sorting** | **HLA-B (-)** | | | | |
| **Allele** | **Clone ID** | **HLA-B** | **HLA-C** | **CIITA** |  |
|  | WT-hESC#1 | WT | -4633 bp | -71 bp; -8/-10 bp |  |
|  | HLA-A11^R^#1 | -3126 bp |  |  |  |
|  | HLA-A11^R^#2 |  |  |  |  |
|  | HLA-A11^R^#3 |  |  |  |  |
|  | HLA-A11^R^#4 |  |  |  |  |
|  | HLA-A11^R^#5 |  |  |  |  |
|  | HLA-A11^R^#6 |  |  |  |  |
|  | HLA-A11^R^#7 | ‘-3107 bp/-3108 bp |  |  |  |
|  | HLA-A11^R^#8 |  |  |  |  |
|  | HLA-A11^R^#9 |  |  |  |  |
|  | HLA-A11^R^#10 |  |  |  |  |
|  | HLA-A11^R^#11 |  |  |  |  |
|  | HLA-A11^R^#12 |  |  |  |  |
|  | HLA-A11^R^#13 |  |  |  |  |
|  | HLA-A11^R^#14 |  |  |  |  |
|  | HLA-A11^R^#15 |  |  |  |  |
|  | HLA-A11^R^#16 |  |  |  |  |
|  | HLA-A11^R^#17 | WT/-3117 bp |  |  |  |
|  | HLA-A11^R^#18 |  |  |  |  |
|  | HLA-A11^R^#19 |  |  |  |  |
|  | HLA-A11^R^#20 |  |  |  |  |
|  | HLA-A11^R^#21 | WT/-3116 bp |  |  |  |
|  | HLA-A11^R^#22 | WT/-3546 bp |  |  |  |
|  | HLA-A11^R^#23 | WT/-3107 bp |  |  |  |
|  | HLA-A11^R^#24 |  |  |  |  |
|  | HLA-A11^R^#25 |  |  |  |  |
|  | HLA-A11^R^#26 | WT/-3322 bp |  |  |  |
|  | HLA-A11^R^#27 |  |  |  |  |
|  | HLA-A11^R^#28 |  |  |  |  |
|  | HLA-A11^R^#29 |  |  |  |  |
|  | HLA-A11^R^#30 |  |  |  |  |
|  | HLA-A11^R^#31 |  |  |  |  |
|  | **HLA-A11^R^#32** | **-3312 bp/-3312 bp** |  |  | **Uesd in study (HLA-A11^R^)** |

**Continued Table S3**

| **Target cell** | **HLA-A,B^R^#1** | | | | |
| --- | --- | --- | --- | --- | --- |
| **Used gRNA(s)** | **HLA-B-gRNA-6, 22, 24** | | | | |
| **Sorting** | **HLA-B (-)** | | | | |
| **Allele** | **Clone ID** | **HLA-B** | **HLA-C** | **CIITA** | **Clone ID** |
|  | HLA-A11^R^#36 | WT/-3321 bp |  |  |  |
|  | HLA-A11^R^#37 | -3110 bp/'-3122 bp |  |  |  |
|  | HLA-A11^R^#38 |  |  |  |  |
|  | HLA-A11^R^#39 |  |  |  |  |
|  | HLA-A11^R^#40 |  |  |  |  |
|  | HLA-A11^R^#41 | WT/-3116 bp |  |  |  |
|  | HLA-A11^R^#42 |  |  |  |  |
|  | HLA-A11^R^#43 | WT/-3711 bp |  |  |  |
|  | HLA-A11^R^#44 |  |  |  |  |
|  | HLA-A11^R^#45 |  |  |  |  |
|  | HLA-A11^R^#46 |  |  |  |  |
|  | HLA-A11^R^#47 |  |  |  |  |
|  | HLA-A11^R^#48 |  |  |  |  |
|  | HLA-A11^R^#49 |  |  |  |  |
|  | HLA-A11^R^#50 | -3109 bp; -3109 bp |  |  |  |
|  | HLA-A11^R^#51 | -3121 bp; -3121 bp |  |  |  |
|  | HLA-A11^R^#52 |  |  |  |  |
|  | HLA-A11^R^#53 |  |  |  |  |
|  | HLA-A11^R^#54 |  |  |  |  |
|  | HLA-A11^R^#55 | WT/- 3147 bp |  |  |  |
|  | HLA-A11^R^#56 |  |  |  |  |
|  | HLA-A11^R^#57 | ‘-3114 bp; -3114 bp |  |  |  |
|  | HLA-A11^R^#58 | WT/-3329 bp |  |  |  |
|  | HLA-A11^R^#59 | WT/3112 bp |  |  |  |
|  | HLA-A11^R^#60 |  |  |  |  |
|  | HLA-A11^R^#61 | WT/-3116 bp |  |  |  |
|  | HLA-A11^R^#62 |  |  |  |  |
|  | Efficiency |  |  |  | 11.29% |

**Continued Table S3**

| **Target cell** | **HLA-A11^R^ hESC** | | |
| --- | --- | --- | --- |
| **Used plasmids** | **iC9-cassette** | **Cas9-GFP** | **hAAVS1-gRNA6** |
| **Screening** | **iC9 (+)** | | |
| **Allele** | **Clone ID** | **Knock in** |  |
|  | iC9-HLA-A11^R^#1 | Yes |  |
|  | iC9-HLA-A11^R^#2 | Yes |  |
|  | iC9-HLA-A11^R^#3 |  |  |
|  | iC9-HLA-A11^R^#4 |  |  |
|  | iC9-HLA-A11^R^#5 | Yes |  |
|  | iC9-HLA-A11^R^#6 | Yes |  |
|  | iC9-HLA-A11^R^#7 | Yes |  |
|  | iC9-HLA-A11^R^#8 | Yes |  |
|  | **iC9-HLA-A11^R^#9** | **Yes** | **Uesd in study (iC9-HLA-A11^R^)** |
|  | iC9-HLA-A11^R^#10 | Yes |  |
|  | iC9-HLA-A11^R^#11 | Yes |  |
|  | iC9-HLA-A11^R^#12 |  |  |
|  | iC9-HLA-A11^R^#13 | Yes |  |
|  | iC9-HLA-A11^R^#14 | Yes |  |
|  | iC9-HLA-A11^R^#15 |  |  |
|  | iC9-HLA-A11^R^#16 |  |  |
|  | iC9-HLA-A11^R^#17 | Yes |  |
|  | iC9-HLA-A11^R^#18 |  |  |
|  | iC9-HLA-A11^R^#19 | Yes |  |
|  | iC9-HLA-A11^R^#20 |  |  |
|  | iC9-HLA-A11^R^#21 |  |  |
|  | iC9-HLA-A11^R^#22 | Yes |  |
|  | iC9-HLA-A11^R^#23 |  |  |
|  | iC9-HLA-A11^R^#24 | Yes |  |
|  | iC9-HLA-A11^R^#25 | Yes |  |
|  | iC9-HLA-A11^R^#26 |  |  |
|  | iC9-HLA-A11^R^#27 | Yes |  |
|  | iC9-HLA-A11^R^#28 |  |  |
|  | iC9-HLA-A11^R^#29 |  |  |
|  | iC9-HLA-A11^R^#30 |  |  |
|  | iC9-HLA-A11^R^#31 |  |  |
|  | iC9-HLA-A11^R^#32 |  |  |
|  | iC9-HLA-A11^R^#33 | Yes |  |
|  | iC9-HLA-A11R#34 |  |  |
|  | iC9-HLA-A11R#35 | Yes |  |

**Continued Table S3**

| **Target cell** | **HLA-A11^R^ hESC** | | |
| --- | --- | --- | --- |
| **Used plasmids** | **iC9-cassette** | **Cas9-GFP** | **hAAVS1-gRNA6** |
| **Screening** | **iC9 (+)** | | |
| **Allele** | **Clone ID** | **Knock in** |  |
|  | iC9-HLA-A11R#36 |  |  |
|  | iC9-HLA-A11R#37 | Yes |  |
|  | iC9-HLA-A11^R^#38 | Yes |  |
|  | iC9-HLA-A11^R^#39 |  |  |
|  | iC9-HLA-A11^R^#40 | Yes |  |
|  | iC9-HLA-A11^R^#41 |  |  |
|  | iC9-HLA-A11^R^#42 | Yes |  |
|  | iC9-HLA-A11^R^#43 |  |  |
|  | iC9-HLA-A11^R^#44 | Yes |  |
|  | iC9-HLA-A11^R^#45 |  |  |
|  | Efficiency |  | 53% |

**Table S4. Karyotype analysis statistics of WT, HLA-A11^R^, and iC9-HLA-A11^R^ hESCs**

|  |  | **Chr No.** | | |  |  |
| --- | --- | --- | --- | --- | --- | --- |
| **Clone name** | **Passage** | **<46** | **46** | **>46** | **Modal No.** | **Modal karyotype** |
| WT | p38 | 0 | 20 | 0 | 46 | 46, XY |
| HLA-A11^R^ | p41 | 0 | 20 | 0 | 46 | 46, XY |
| iC9-HLA-A11^R^ | p54 | 0 | 20 | 0 | 46 | 46, XY |

**Table S5. HLA haplotype information of hESCs, PBMCs, and hu-mice used in this study**

|  | **HLA-A** | **HLA-B** | **HLA-C** | **DRB1** | **DQB1** |
| --- | --- | --- | --- | --- | --- |
| WT hESC | A*11:01:01:01 | B*51:01:01:01 | C*14:02:01:01 | DRB1*09:01:02 | DQB1*03:01:01 |
|  | A*11:01:01:01 | B*51:01:01 | C*14:02:01:01 | DRB1*11:01:01 | DQB1*03:03:02 |
| T/NK | A*11:01 | B*35:05 | C*04:01 | DRB1*14:04 | DQB1*05:01 |
|  | A*11:01 | B*52:01 | C*07:02 | DRB1*15:02 | DQB1*05:03 |
| Hu-mice | A*02:01 | B*13:01 | C*03:04 | DRB1*12:01:01G | DQB1*03:01 |
|  | A*11:01 | B*48:01 | C*08:01 | DRB1*12:02 | DQB1*03:03 |

**Table S6. Summary of potential off-target information for HLA-A11^R^ hESCs by WGS and confirmed by NGS**

|  | **Off-target gene** | **Off-target region** | **Strand** | **Off-target position** | **sgRNA** | **Off-target sequence** | **Numbers of mismatch base** | **Edied on off-target sites** | **Analyzed cell line** | **WT sequence info of the mutation site** | **mutation** | **number of Altered reads compared to WT** | **WT reads** | **NGS confirmatIon %** |
| --- | --- | --- | --- | --- | --- | --- | --- | --- | --- | --- | --- | --- | --- | --- |
| Potential off-target sites based on gRNAs | SPATA2P1-RN7SKP6 | intergenic | + | chr13:56282307-56282330 | HLA-B-gRNA-22 | TGCTTTcCActgTCcTGAGTAGA | 5 | chr13:56282310 | HLA-A11^R^#32* | TC | T | 2 | 13 | 0/2186828 (0.0%) |
|  | TANGO6 | downstream_gene | + | chr16:68846873-68846896 | HLA-C-gRNA-7 | GTAtcGaGAAACTCAaCAaGAGG | 5 | chr16:68846893 | HLA-A11^R^#32* | C | CTT | 2 | 17 | 0/267738 (0.0%) |
|  | PDK3 | intronic | - | chrX:24532881-24532904 | HLA-B-gRNA-22 | TGgaTTgCAGATTCtTGgGTTAG | 5 | chrX:24532893 | HLA-A11^R^#7 | TA | T | 2 | 21 | ~ |
|  | PASD1 | intronic | - | chrX:151603170-151603193 | HLA-B-gRNA-22 | TGCTTTACAGgTTtAgGAtaTAG | 5 | chrX:151603174 | HLA-A11^R^#7 | GT | G | 2 | 9 | ~ |
|  | JARID2 | intronic | - | chr6:15437349-15437372 | CIITA-gRNA-5 | aTtCAgCTGGaCATAaAAGGGG | 5 | chr6:15437363 | HLA-A11^R^#7 | CTT | C | 2 | 30 | ~ |
|  | CTC-439O9.3 | intronic | - | chr19:31200250-31200273 | HLA-B-gRNA-22 | TGCTaTACAtATcCATGtGcAGG | 5 | chr19:31200250 | HLA-A11^R^#37 | G | GA | 3 | 25 | ~ |
|  | CTC-439O9.3 | intronic | - | chr20:58178054-58178077 | HLA-C-gRNA-14 | CAAGGGCgGTtTCTGggaTGAAG | 5 | chr20:58178076 | HLA-A11^R^#37 | AA | CC | 4 | 37 | ~ |
|  | MIR4272-RP11-85I21.1 | intergenic | - | chr3:67231596-67231619 | HLA-B-gRNA-6 | GTCCtGgGaCCcCAtGGAGGAGA | 5 | chr3:67231616 | HLA-A11^R^#37 | TG | T | 2 | 16 | ~ |
|  | SP100 | upstream_gene | + | chr2:230499883-230499906 | Q78-HLA-C-gRNA-22 | TATCTGAcaATtctCTCCAGAGA | 5 | chr2:230499904 | iC9-HLA-A11^R^#9* | CC | GG | 4 | 43 | 5/2617583 (0.0%) |
| Potential off-target sites related with cancer independent on gRNAs | Metazoa-SRP | exonic | ~ | ~ | ~ | ~ | ~ | chr1:193236963 | HLA-A11^R^#32* | AGAC | A | 1 | 14 | 6/58264 (0.0%) |
|  | RARA | exonic | ~ | ~ | ~ | ~ | ~ | chr17:40343077 | HLA-A11^R^#32* | TG | T | 2 | 12 | 0/2849193 (0.0%) |
|  | ACSL6 | exonic | ~ | ~ | ~ | ~ | ~ | chr5:131994059 | HLA-A11^R^#32* | TTG | T | 2 | 14 | 0/2605769 (0.0%) |
|  | PLCG1 | exonic | ~ | ~ | ~ | ~ | ~ | chr20:41163236 | iC9-HLA-A11^R^#9* | CCT | C | 3 | 40 | 51/3719128 (0.0%) |
| Note. * means the HLA-A11^R^ hESC uesd in this study |  |  |  |  |  |  |  |  |  |  |  |  |  |  |

**Table S7. Coverage percentage by HLA-A, HLA-B, and HLA-C in the Chinese population**

| **Rank** | **HLA-A** | **Count** | **frequency** | **Cumulative HLA-A** |
| --- | --- | --- | --- | --- |
| 1 | A*11:01 | 71884 | 21.14297 | 21.14297 |
| 2 | A*24:02 | 52932 | 15.56869 | 36.71166 |
| 3 | A*02:01 | 41784 | 12.28977 | 49.00143 |
| 4 | A*02:07 | 28911 | 8.50349 | 57.50492 |
| 5 | A*33:03 | 26965 | 7.93112 | 65.43604 |
| 6 | A*30:01 | 18475 | 5.43398 | 70.87002 |
| 7 | A*02:06 | 17678 | 5.19956 | 76.06958 |
| 8 | A*02:03 | 11887 | 3.49628 | 79.56586 |
| 9 | A*01:01 | 11572 | 3.40363 | 82.96949 |
| 10 | A*31:01 | 11215 | 3.29863 | 86.26812 |
| 11 | A*03:01 | 10204 | 3.00126 | 89.26938 |
| 12 | A*26:01 | 9509 | 2.79685 | 92.06623 |
| *13* | A*11:02 | 6517 | 1.91682 | 93.98305 |
| 14 | A*32:01 | 4349 | 1.27916 | 95.26221 |

**Continued Table S7**

| **Rank** | **HLA-C** | **Count** | **frequency** | **Cumulative HLA-C** |
| --- | --- | --- | --- | --- |
| 1 | C*01:02 | 52897 | 15.5584 | 15.5584 |
| 2 | C*07:02 | 51515 | 15.15192 | 30.71032 |
| 3 | C*03:04 | 33852 | 9.95676 | 40.66708 |
| 4 | C*06:02 | 30355 | 8.9282 | 49.59528 |
| 5 | C*08:01 | 28771 | 8.46231 | 58.05759 |
| 6 | C*03:03 | 24199 | 7.11756 | 65.17515 |
| 7 | C*04:01 | 20317 | 5.97576 | 71.15091 |
| 8 | C*03:02 | 19877 | 5.84635 | 76.99726 |
| 9 | C*14:02 | 14412 | 4.23895 | 81.23621 |
| 10 | C*15:02 | 11491 | 3.37981 | 84.61602 |
| 11 | C*12:02 | 10829 | 3.18509 | 87.80111 |
| 12 | C*12:03 | 6541 | 1.92388 | 89.72499 |
| 13 | C*14:03 | 3579 | 1.05268 | 90.77767 |
| 14 | C*04:03 | 3426 | 1.00768 | 91.78535 |
| 15 | C*08:22 | 3214 | 0.94532 | 92.73067 |
| 16 | C*05:01 | 3017 | 0.88738 | 93.61805 |
| 17 | C*07:04 | 3011 | 0.88561 | 94.50366 |
| 18 | C*07:06 | 2757 | 0.81091 | 95.31457 |

**Continued Table S7**

| **Rank** | **HLA-B** | **Count** | **frequency** | **Cumulative HLA-B** |
| --- | --- | --- | --- | --- |
| 1 | B*46:01 | 34749 | 10.22059 | 10.22059 |
| 2 | B*40:01 | 33849 | 9.95588 | 20.17647 |
| 3 | B*58:01 | 19955 | 5.86929 | 26.04576 |
| 4 | B*13:02 | 19614 | 5.76899 | 31.81475 |
| 5 | B*51:01 | 19163 | 5.63634 | 37.45109 |
| 6 | B*13:01 | 17044 | 5.01309 | 42.46418 |
| 7 | B*15:01 | 16716 | 4.91662 | 47.3808 |
| 8 | B*15:02 | 12051 | 3.54452 | 50.92532 |
| 9 | B*40:06 | 10989 | 3.23215 | 54.15747 |
| 10 | B*54:01 | 10332 | 3.03891 | 57.19638 |
| 11 | B*52:01 | 9704 | 2.8542 | 60.05058 |
| 12 | B*35:01 | 9698 | 2.85244 | 62.90302 |
| 13 | B*44:03 | 8940 | 2.62949 | 65.53251 |
| 14 | B*38:02 | 8884 | 2.61302 | 68.14553 |
| 15 | B*48:01 | 8369 | 2.46154 | 70.60707 |
| 16 | B*55:02 | 8335 | 2.45154 | 73.05861 |
| 17 | B*07:02 | 7426 | 2.18418 | 75.24279 |
| 18 | B*40:02 | 6561 | 1.92976 | 77.17255 |
| 19 | B*15:11 | 6227 | 1.83152 | 79.00407 |
| 20 | B*39:01 | 6066 | 1.78417 | 80.78824 |
| 21 | B*37:01 | 4579 | 1.3468 | 82.13504 |
| 22 | B*15:18 | 4574 | 1.34533 | 83.48037 |
| 23 | B*35:03 | 3760 | 1.10591 | 84.58628 |
| 24 | B*57:01 | 3713 | 1.09209 | 85.67837 |
| 25 | B*51:02 | 3592 | 1.0565 | 86.73487 |
| 26 | B*44:02 | 3323 | 0.97738 | 87.71225 |
| 27 | B*27:04 | 3210 | 0.94415 | 88.6564 |
| 28 | B*08:01 | 2931 | 0.86208 | 89.51848 |
| 29 | B*15:27 | 2752 | 0.80944 | 90.32792 |
| 30 | B*27:05 | 2603 | 0.76561 | 91.09353 |
| 31 | B*67:01 | 2539 | 0.74679 | 91.84032 |
| 32 | B*07:05 | 2489 | 0.73208 | 92.5724 |
| 33 | B*15:25 | 2123 | 0.62443 | 93.19683 |
| 34 | B*50:01 | 2108 | 0.62002 | 93.81685 |
| 35 | B*56:01 | 1761 | 0.51796 | 94.33481 |
| 36 | B*38:01 | 1662 | 0.48884 | 94.82365 |
| 37 | B*18:01 | 1357 | 0.39913 | 95.22278 |

**Table S8. Top 20 HLA-A allele frequencies by geographical region in China**

| **Allele** | **Num. China** | **%** | **Num. North** | **%** | **Num. South** | **%** | **Num. North China** | **%** | **Num. Northeast** | **%** | **Num. East China** | **%** | **Num. Central China** | **%** | **Num. South China** | **%** | **Num. Southwest** | **%** | **Num. Northwest** | **%** |
| --- | --- | --- | --- | --- | --- | --- | --- | --- | --- | --- | --- | --- | --- | --- | --- | --- | --- | --- | --- | --- |
| [A*11:01](http://cmms.dnaday.cn/geneReport.do?method=alleleMapArea&lang=zh&cate=1&allele=A*11:01&n=15.94446&s=25.35486&mn=16.12387&en=14.88834&me=20.28671&mm=22.82253&ms=28.12081&ws=27.2289&wn=18.51968) | 71884 | 21.143 | 24263 | 15.944 | 47621 | 25.35 | 8971.00 | 16.12 | 3720.00 | 14.89 | 20746.00 | 20.29 | 11152.00 | 22.82 | 10493.00 | 28.12 | 11465.00 | 27.23 | 5337.00 | 18.52 |
| [A*24:02](http://cmms.dnaday.cn/geneReport.do?method=alleleMapArea&lang=zh&cate=1&allele=A*24:02&n=15.33528&s=15.75781&mn=15.65297&en=15.5367&me=15.62524&mm=15.78053&ms=14.90325&ws=15.68423&wn=15.56666) | 52932 | 15.5687 | 23336 | 15.335 | 29596 | 15.76 | 8709.00 | 15.65 | 3882.00 | 15.54 | 15979.00 | 15.63 | 7711.00 | 15.78 | 5561.00 | 14.90 | 6604.00 | 15.68 | 4486.00 | 15.57 |
| [A*02:01](http://cmms.dnaday.cn/geneReport.do?method=alleleMapArea&lang=zh&cate=1&allele=A*02:01&n=15.34316&s=9.81589&mn=15.82731&en=16.6133&me=12.77185&mm=11.28029&ms=7.34309&ws=8.30998&wn=13.93226) | 41784 | 12.2898 | 23348 | 15.343 | 18436 | 9.82 | 8806.00 | 15.83 | 4151.00 | 16.61 | 13061.00 | 12.77 | 5512.00 | 11.28 | 2740.00 | 7.34 | 3499.00 | 8.31 | 4015.00 | 13.93 |
| [A*02:07](http://cmms.dnaday.cn/geneReport.do?method=alleleMapArea&lang=zh&cate=1&allele=A*02:07&n=5.49575&s=10.94038&mn=5.75146&en=5.44305&me=8.30498&mm=9.9562&ms=11.56938&ws=11.43305&wn=6.46124) | 28911 | 8.50349 | 8363 | 5.4958 | 20548 | 10.94 | 3200.00 | 5.75 | 1360.00 | 5.44 | 8493.00 | 8.30 | 4865.00 | 9.96 | 4317.00 | 11.57 | 4814.00 | 11.43 | 1862.00 | 6.46 |
| [A*33:03](http://cmms.dnaday.cn/geneReport.do?method=alleleMapArea&lang=zh&cate=1&allele=A*33:03&n=6.85014&s=8.80693&mn=5.87189&en=7.07196&me=9.51361&mm=7.36125&ms=9.61569&ws=7.54999&wn=6.37796) | 26965 | 7.93112 | 10424 | 6.8501 | 16541 | 8.81 | 3267.00 | 5.87 | 1767.00 | 7.07 | 9729.00 | 9.51 | 3597.00 | 7.36 | 3588.00 | 9.62 | 3179.00 | 7.55 | 1838.00 | 6.38 |
| [A*30:01](http://cmms.dnaday.cn/geneReport.do?method=alleleMapArea&lang=zh&cate=1&allele=A*30:01&n=7.32461&s=3.90218&mn=6.40569&en=7.33211&me=6.50571&mm=5.54805&ms=2.64244&ws=2.85233&wn=5.30224) | 18475 | 5.43398 | 11146 | 7.3246 | 7329 | 3.90 | 3564.00 | 6.41 | 1832.00 | 7.33 | 6653.00 | 6.51 | 2711.00 | 5.55 | 986.00 | 2.64 | 1201.00 | 2.85 | 1528.00 | 5.30 |
| [A*02:06](http://cmms.dnaday.cn/geneReport.do?method=alleleMapArea&lang=zh&cate=1&allele=A*02:06&n=6.54457&s=4.10983&mn=6.7364&en=7.3241&me=5.1103&mm=4.67624&ms=3.6233&ws=3.76431&wn=5.73253) | 17678 | 5.19956 | 9959 | 6.5446 | 7719 | 4.11 | 3748.00 | 6.74 | 1830.00 | 7.32 | 5226.00 | 5.11 | 2285.00 | 4.68 | 1352.00 | 3.62 | 1585.00 | 3.76 | 1652.00 | 5.73 |
| [A*02:03](http://cmms.dnaday.cn/geneReport.do?method=alleleMapArea&lang=zh&cate=1&allele=A*02:03&n=1.94451&s=4.75354&mn=2.00942&en=1.793&me=2.81526&mm=3.2437&ms=7.71292&ws=5.53603&wn=2.24859) | 11887 | 3.49628 | 2959 | 1.9445 | 8928 | 4.75 | 1118.00 | 2.01 | 448.00 | 1.79 | 2879.00 | 2.82 | 1585.00 | 3.24 | 2878.00 | 7.71 | 2331.00 | 5.54 | 648.00 | 2.25 |
| [A*01:01](http://cmms.dnaday.cn/geneReport.do?method=alleleMapArea&lang=zh&cate=1&allele=A*01:01&n=4.89709&s=2.19361&mn=5.12599&en=4.49051&me=3.00106&mm=3.03291&ms=1.46594&ws=2.34171&wn=5.25366) | 11572 | 3.40363 | 7452 | 4.8971 | 4120 | 2.19 | 2852.00 | 5.13 | 1122.00 | 4.49 | 3069.00 | 3.00 | 1482.00 | 3.03 | 547.00 | 1.47 | 986.00 | 2.34 | 1514.00 | 5.25 |
| [A*31:01](http://cmms.dnaday.cn/geneReport.do?method=alleleMapArea&lang=zh&cate=1&allele=A*31:01&n=4.03162&s=2.70475&mn=3.94155&en=4.27439&me=3.37949&mm=3.24165&ms=1.85989&ws=2.64095&wn=3.84482) | 11215 | 3.29863 | 6135 | 4.0316 | 5080 | 2.70 | 2193.00 | 3.94 | 1068.00 | 4.27 | 3456.00 | 3.38 | 1584.00 | 3.24 | 694.00 | 1.86 | 1112.00 | 2.64 | 1108.00 | 3.84 |
| [A*03:01](http://cmms.dnaday.cn/geneReport.do?method=alleleMapArea&lang=zh&cate=1&allele=A*03:01&n=4.44826&s=1.8289&mn=4.74316&en=4.34243&me=2.48377&mm=2.63384&ms=1.42574&ws=2.08284&wn=4.31675) | 10204 | 3.00126 | 6769 | 4.4483 | 3435 | 1.83 | 2639.00 | 4.74 | 1085.00 | 4.34 | 2540.00 | 2.48 | 1287.00 | 2.63 | 532.00 | 1.43 | 877.00 | 2.08 | 1244.00 | 4.32 |
| [A*26:01](http://cmms.dnaday.cn/geneReport.do?method=alleleMapArea&lang=zh&cate=1&allele=A*26:01&n=3.20558&s=2.46568&mn=3.31069&en=3.0377&me=2.61578&mm=2.87123&ms=2.03677&ws=2.57683&wn=3.418) | 9509 | 2.79685 | 4878 | 3.2056 | 4631 | 2.47 | 1842.00 | 3.31 | 759.00 | 3.04 | 2675.00 | 2.62 | 1403.00 | 2.87 | 760.00 | 2.04 | 1085.00 | 2.58 | 985.00 | 3.42 |
| [A*11:02](http://cmms.dnaday.cn/geneReport.do?method=alleleMapArea&lang=zh&cate=1&allele=A*11:02&n=0.9923&s=2.66588&mn=1.02628&en=1.01657&me=2.076&mm=1.91961&ms=3.8109&ws=2.10896&wn=1.11389) | 6517 | 1.91682 | 1510 | 0.9923 | 5007 | 2.67 | 571.00 | 1.03 | 254.00 | 1.02 | 2123.00 | 2.08 | 938.00 | 1.92 | 1422.00 | 3.81 | 888.00 | 2.11 | 321.00 | 1.11 |
| [A*32:01](http://cmms.dnaday.cn/geneReport.do?method=alleleMapArea&lang=zh&cate=1&allele=A*32:01&n=1.85448&s=0.81302&mn=1.77037&en=1.69295&me=1.28198&mm=1.35069&ms=0.46363&ws=0.76474&wn=1.64828) | 4349 | 1.27916 | 2822 | 1.8545 | 1527 | 0.81 | 985.00 | 1.77 | 423.00 | 1.69 | 1311.00 | 1.28 | 660.00 | 1.35 | 173.00 | 0.46 | 322.00 | 0.76 | 475.00 | 1.65 |
| [A*29:01](http://cmms.dnaday.cn/geneReport.do?method=alleleMapArea&lang=zh&cate=1&allele=A*29:01&n=0.97193&s=0.73742&mn=0.83935&en=0.77243&me=0.84487&mm=0.79404&ms=0.83615&ws=0.84786&wn=0.98203) | 2864 | 0.84238 | 1479 | 0.9719 | 1385 | 0.74 | 467.00 | 0.84 | 193.00 | 0.77 | 864.00 | 0.84 | 388.00 | 0.79 | 312.00 | 0.84 | 357.00 | 0.85 | 283.00 | 0.98 |
| [A*68:01](http://cmms.dnaday.cn/geneReport.do?method=alleleMapArea&lang=zh&cate=1&allele=A*68:01&n=1.02318&s=0.51539&mn=1.00471&en=0.90451&me=0.62974&mm=0.70604&ms=0.36179&ws=0.68874&wn=1.13124) | 2525 | 0.74267 | 1557 | 1.0232 | 968 | 0.52 | 559.00 | 1.00 | 226.00 | 0.90 | 644.00 | 0.63 | 345.00 | 0.71 | 135.00 | 0.36 | 290.00 | 0.69 | 326.00 | 1.13 |
| [A*02:10](http://cmms.dnaday.cn/geneReport.do?method=alleleMapArea&lang=zh&cate=1&allele=A*02:10&n=0.59209&s=0.27207&mn=0.58593&en=0.54831&me=0.40875&mm=0.39293&ms=0.1876&ws=0.247&wn=0.57256) | 1412 | 0.41531 | 901 | 0.5921 | 511 | 0.27 | 326.00 | 0.59 | 137.00 | 0.55 | 418.00 | 0.41 | 192.00 | 0.39 | 70.00 | 0.19 | 104.00 | 0.25 | 165.00 | 0.57 |
| [A*02:05](http://cmms.dnaday.cn/geneReport.do?method=alleleMapArea&lang=zh&cate=1&allele=A*02:05&n=0.52769&s=0.17836&mn=0.52302&en=0.55631&me=0.26989&mm=0.29265&ms=0.10184&ws=0.19475&wn=0.58644) | 1138 | 0.33472 | 803 | 0.5277 | 335 | 0.18 | 291.00 | 0.52 | 139.00 | 0.56 | 276.00 | 0.27 | 143.00 | 0.29 | 38.00 | 0.10 | 82.00 | 0.19 | 169.00 | 0.59 |
| [A*23:01](http://cmms.dnaday.cn/geneReport.do?method=alleleMapArea&lang=zh&cate=1&allele=A*23:01&n=0.40218&s=0.16186&mn=0.50865&en=0.28416&me=0.20926&mm=0.19442&ms=0.09648&ws=0.19237&wn=0.47193) | 916 | 0.26942 | 612 | 0.4022 | 304 | 0.16 | 283.00 | 0.51 | 71.00 | 0.28 | 214.00 | 0.21 | 95.00 | 0.19 | 36.00 | 0.10 | 81.00 | 0.19 | 136.00 | 0.47 |
| [A*24:20](http://cmms.dnaday.cn/geneReport.do?method=alleleMapArea&lang=zh&cate=1&allele=A*24:20&n=0.24972&s=0.2561&mn=0.23365&en=0.24414&me=0.27771&mm=0.26604&ms=0.23048&ws=0.20187&wn=0.29495) | 861 | 0.25324 | 380 | 0.2497 | 481 | 0.26 | 130.00 | 0.23 | 61.00 | 0.24 | 284.00 | 0.28 | 130.00 | 0.27 | 86.00 | 0.23 | 85.00 | 0.20 | 85.00 | 0.29 |

**Table S9. Summary of 14 HLA-A alleles in each geographical region in the world**

| **Summary** | **Australia** | **Europe** | **North America** | **North-East Asia** | **North African** | **Oceania** | **South Asia** | **South-Central America** | **South-East Asia** | **Sub-Saharan Africa** | **Western Asia** |
| --- | --- | --- | --- | --- | --- | --- | --- | --- | --- | --- | --- |
| A*11:01 | 0.1177 | 0.0527 | 0.070162701 | 0.081233855 | 0.01023853 | 0.217743945 | 0.142147195 | 0.038162726 | 0.228710739 | 0.026449546 | 0.049403769 |
| A*24:02 | 0.213700368 | 0.088442743 | 0.098805028 | 0.284950976 | 0.045241228 | 0.333442387 | 0.115520176 | 0.13971485 | 0.090458967 | 0.022691234 | 0.039672257 |
| A*02:01 | 0.160216912 | 0.274738563 | 0.170504885 | 0.118675772 | 0.141622807 | 0.055757202 | 0.048514256 | 0.147347993 | 0.137125714 | 0.107197313 | 0.082639868 |
| A*02:07 | 0.003298462 | 0.000238472 | 0.005980616 | 0.031261701 | 0 | 0.009512821 | 0 | 0.000483821 | 0.106671983 | 0.0025 | 0.000149991 |
| A*33:03 | 0.002455607 | 0.003720049 | 0.030121203 | 0.081601959 | 0.030579394 | 0.122739269 | 0.099366209 | 0.011250315 | 0.097137311 | 0.019455212 | 0.00491011 |
| A*30:01 | 0.006184615 | 0.014526046 | 0.024990876 | 0.008096047 | 0.060465652 | 0.00881051 | 0.016285783 | 0.021975422 | 0.032433538 | 0.055225675 | 0.026 |
| A*02:06 | 0 | 0.001903104 | 0.013353413 | 0.081404841 | 0 | 0.05829561 | 0.021699599 | 0.001011976 | 0.042355898 | 0.003671151 | 0.000954128 |
| A*02:03 | 0 | 0.00 | 0.005507615 | 0.001549598 | 0.002958683 | 0.035665392 | 0.011150613 | 0.000759641 | 0.051934889 | 0.005141414 | 0.000340631 |
| A*01:01 | 0.067733598 | 0.151214361 | 0.109850644 | 0.01530976 | 0.076851553 | 0.028687198 | 0.127635903 | 0.070778831 | 0.015118231 | 0.063771576 | 0.153184971 |
| A*31:01 | 0.0145 | 0.023379433 | 0.028740993 | 0.06988176 | 0.015596418 | 0.027007856 | 0.038022236 | 0.063306618 | 0.018187139 | 0.008581274 | 0.019382119 |
| A*03:01 | 0.045015152 | 0.146829453 | 0.098939261 | 0.018041928 | 0.058434211 | 0.012261468 | 0.063555228 | 0.066294456 | 0.013868386 | 0.056128713 | 0.031004915 |
| A*26:01 | 0.009070769 | 0.031522964 | 0.028544812 | 0.062089126 | 0.015146185 | 0.022791785 | 0.040108928 | 0.02412381 | 0.022315443 | 0.075392018 | 0.072269627 |
| A*11:02 | 0 | 0.00 | 0.001590767 | 0.002147045 | 0 | 0.05 | 0.000578704 | 0 | 0.031519515 | 0 | 0.0000540 |
| A*32:01 | 0.015131213 | 0.035557247 | 0.027615632 | 0.004708971 | 0.036392864 | 0.002061201 | 0.030217271 | 0.022456981 | 0.00615786 | 0.019150694 | 0.038229696 |
| Total | 0.655006696 | 0.824914809 | 0.714708446 | 0.86095334 | 0.493527524 | 0.984776642 | 0.7548021 | 0.607667438 | 0.893995614 | 0.465355818 | 0.5181961 |

Note: Allele Frequency = Total number of copies of the allele in the population sample (Alleles/2n)
